# Ketotifen directly modifies the fibrotic response of human skin fibroblasts

**DOI:** 10.1038/s41598-024-57776-7

**Published:** 2024-03-25

**Authors:** Edwin Leong, Haya Al-Bitar, Jean S. Marshall, Michael Bezuhly

**Affiliations:** 1 Department of Pathology, Dalhousie University, 5850 College Street, Room 7-C, PO BOX 15000, Halifax, NS B3H 4R2 Canada; 2 Department of Microbiology and Immunology, Dalhousie University, Halifax, Canada; 3 Beatrice Hunter Cancer Research Institute, Halifax, Canada; 4 Division of Plastic Surgery, Izaak Walton Killam Health Centre, 5850/5980 University Avenue, PO Box 9700, Halifax, NS B3K 6R8 Canada; 5 Department of Surgery, Dalhousie University, Halifax, Canada

**Keywords:** Mechanisms of disease, Experimental models of disease, Translational research, Skin diseases

## Abstract

Fibrosis is a destructive, end-stage disease process. In the skin, it is associated with systemic sclerosis and scarring with considerable health burden. Ketotifen is a clinical antihistamine and mast cell stabilizer. Studies have demonstrated mast cell-dependent anti-fibrotic effects of ketotifen but direct effects on fibroblasts have not been determined. Human dermal fibroblasts were treated with pro-fibrotic transforming growth factor-β1 (TGFβ) followed by ketotifen or control treatments to determine direct effects on fibrotic fibroblasts. Ketotifen impaired TGFβ-induced α-smooth muscle actin gene and protein responses and decreased cytoskeletal- and contractility-associated gene responses associated with fibrosis. Ketotifen reduced Yes-associated protein phosphorylation, transcriptional coactivator with PDZ binding motif transcript and protein levels, and phosphorylation of protein kinase B. In a fibroblast-populated collagen gel contraction assay, ketotifen reduced the contractile activity of TGFβ-activated fibroblasts. In a murine model of bleomycin-induced skin fibrosis, collagen density and dermal thickness were significantly decreased in ketotifen-treated mice supporting in vitro findings. These results support a novel, direct anti-fibrotic activity of ketotifen, reducing pro-fibrotic phenotypic changes in fibroblasts and reducing collagen fibres in fibrotic mouse skin. Together, these findings suggest novel therapeutic potential and a novel mechanism of action for ketotifen in the context of fibrosis.

## Introduction

Dermal fibrosis contributes to debilitating conditions including systemic sclerosis, burn wounds, surgical scars, and chronic graft-versus-host disease. Fibrosis is characterized by excessive extracellular matrix (ECM) deposition^1,2^. Accumulation of ECM in the skin has considerable impacts including reduced mobility, impaired function, and psychological burdens associated with scarring. Fibroblasts are the principal cells dysregulated during fibrosis and exhibit considerable heterogeneity based on tissue site, although their functions remain well-conserved^3,4^. Fibroblasts are essential to maintain ECM composition and facilitate tissue repair by producing proteins such as collagen. Pro-fibrotic mediators like transforming growth factor-β1 (TGFβ1) induce fibroblast subpopulations to terminally differentiate into contractile α-smooth muscle actin (αSMA)-positive myofibroblasts, promoting ECM synthesis and matrix re-organization^5,6^. These physiological events are tightly regulated in wound repair, and their dysregulation and sustained activation result in fibrosis^7^.

Multiple signals promote fibrosis, including mechano-transduction within the ECM^8^. Activation of fibroblasts by TGFβ1 promotes a contractile phenotype, thus eliciting mechanical forces on the ECM during fibrosis. The processes involved in mechano-transduction are prevalent in hypertrophic skin scarring^9^ and reduced in dermal fibroblasts derived from aged atrophic skin^10^. Interactions between integrins, focal adhesion molecules, and other mechanical cues are one mechanism by which latent TGFβ1 complexes are activated^11^. This leads to further increased differentiation of fibroblasts into more contractile myofibroblasts, thus establishing a feed-forward loop^12^. In this setting, αSMA and actin-binding proteins such as calponins and transgelins that influence cytoskeletal structure are modified^13–15^. Mechano-transduction is therefore critical in directing the microenvironment towards perpetuation or resolution of fibrosis^16,17^. Mechano-signals drive pro-fibrotic changes through effector molecules of the Hippo pathway, Yes-associated protein (YAP) and transcriptional coactivator with PDZ-binding motif (TAZ)^18,19^. YAP and TAZ regulate organ size and shape during embryogenesis and tissue regeneration, and are tightly controlled^20,21^. In the presence of pro-fibrotic stimuli such as mechano-transduction and TGFβ1, YAP/TAZ signaling promotes contractility and differentiation of activated fibroblasts^19,22^.

Ketotifen, a well-known mast cell stabilizer and H1 receptor antagonist, has previously been investigated in fibrosis. Studies have attributed the anti-fibrotic effects of ketotifen to the drug’s activity on resident mast cells. Ketotifen treatment was associated with decreased αSMA expression in models of skin wound repair^23^ and knee arthrofibrosis^24^. Furthermore, ketotifen reduced the inflammatory profile and severity of contractures in arthrofibrosis models^25^. While these studies support a role for mast cells as regulators of fibrosis and its resolution, the impact of ketotifen directly on fibroblasts, the principal effectors in fibrosis have not been investigated. We examined the ability of ketotifen to modify the fibrotic response in human dermal fibroblasts.

## Results

### Ketotifen selectively decreased α-smooth muscle actin expression in TGFβ1-treated dermal fibroblasts

To assess the effect of ketotifen on unstimulated fibroblasts, adult human dermal fibroblasts (HDFa and WS1 cells) were incubated with or without the drug for 48 h. To assess the effect of ketotifen on activated fibroblasts, HDFa and WS1 cells were incubated with TGFβ1 (10 ng/mL) for 48 h to induce a pro-fibrotic state. Ketotifen was added to the TGFβ1-activated fibroblasts during the final 24 h, concurrently with continued TGFβ1 treatment. Levels of transcription of *ACTA2*, the gene that encodes αSMA, were determined to assess the effect of ketotifen on differentiation towards a myofibroblast phenotype.

**Figure 1:**
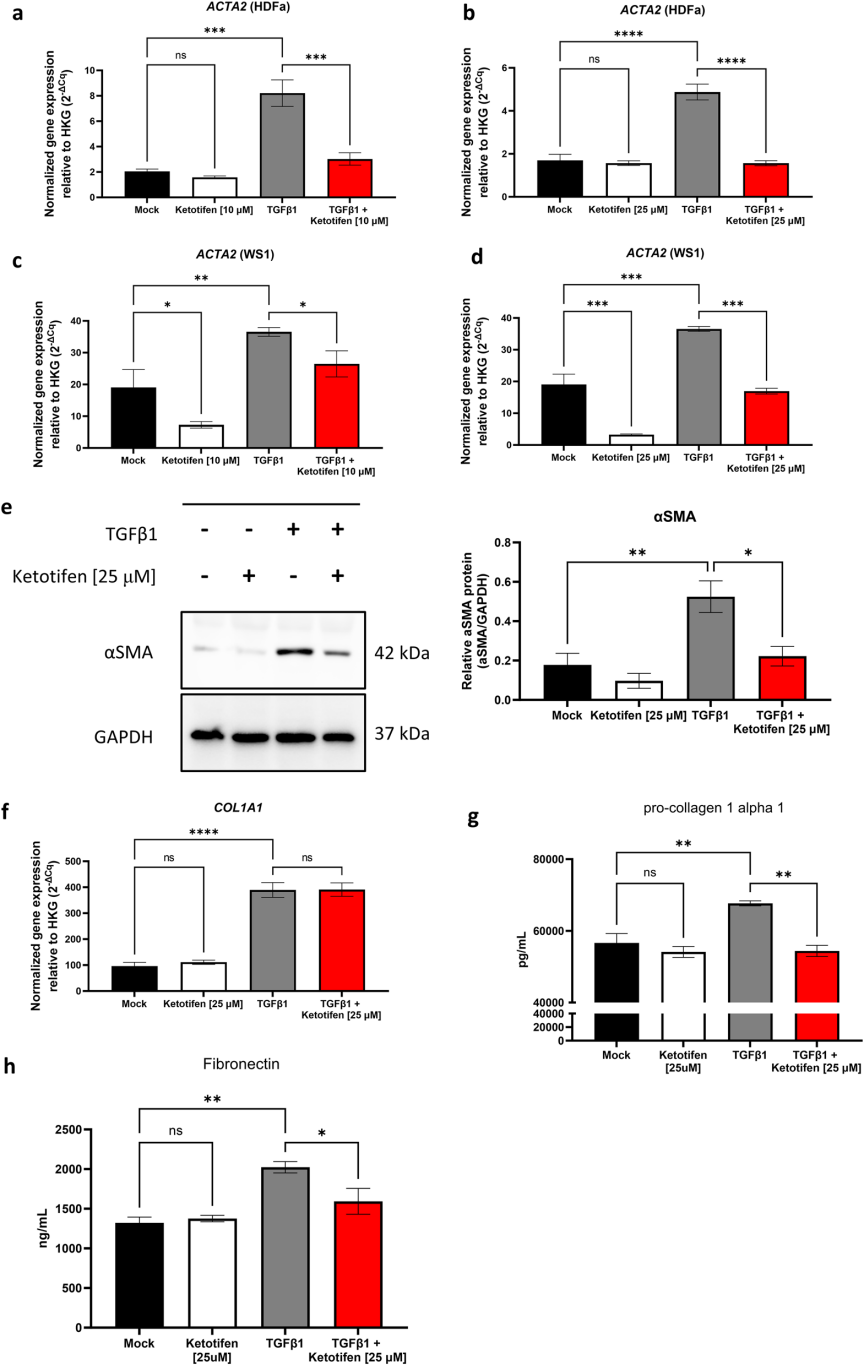
αSMA gene expression and protein levels are reduced in TGFβ1-activated fibroblasts following ketotifen treatment. (**A**, **B**) HDFa and (**C**, **D**) WS1 fibroblasts were treated for 48 h with DMEM supplemented with 10% FBS as mock, 10 μM or 25 μM ketotifen, 10 ng/mL TGFβ1, or 10 ng/mL TGFβ1 with ketotifen added during the final 24 h. Gene expression of αSMA (*ACTA2*) was measured using RT-qPCR and normalized to housekeeping gene *HPRT*. HDFa cells were treated for 48 h under the same conditions and probed for αSMA protein by western blot. (**E**) A representative image of the blots is shown (left) and semi-quantitative assessments plotted (right) using GAPDH as loading control. Full-length blots are available in Supplementary Fig. 4. (**F**) *COL1A1* was measured in HDFa cells treated with conditions using 25 μM ketotifen. Protein levels of pro-collagen 1α1 (**G**) and fibronectin (**H**) were determined by ELISA staining kits. Data shown as mean ± SEM. n = 3–6 per treatment condition. *p < 0.05; **p < 0.01; ***p < 0.001; ****p < 0.0001. *αSMA* alpha-smooth muscle actin, *GAPDH* glyceraldehyde 3-phosphate dehydrogenase, *HDFa* human dermal fibroblasts (adult), *SEM* standard error of the mean, *TGFβ1* transforming growth factor-beta.

HDFa cells were unaffected by ketotifen treatments at rest, compared to untreated controls (Fig. 1A,B). TGFβ1-activated HDFa cells demonstrated increased *ACTA2* expression. These responses were reduced in TGFβ1 activated cells treated with ketotifen (Fig. 1A,B). An alternate dermal fibroblast cell line, WS1, derived from normal skin during gestation, showed a considerable decrease in *ACTA2* with ketotifen treatment at rest (Fig. 1C,D). As in HDFa cells, upregulation of *ACTA2* gene expression in TGFβ1-activated WS1 fibroblasts was inhibited by ketotifen. Since adult primary HDFa cells were the more physiologically relevant model, further experiments focused on these cells.

The impact of a range of doses of ketotifen on TGFβ1-activated HDFa cells was examined. A trend towards decreasing *ACTA2* expression was observed with increasing ketotifen concentrations (Supplementary Fig. 1). To evaluate potential cell toxicity of ketotifen treatments, HDFa cells were examined by Annexin-V and 7-Aminoactinomycin D (7-AAD) staining. No differences were observed in cell viability or Annexin-V positivity between ketotifen treated and control groups of fibroblasts (Supplementary Fig. 2).

Given these responses, intermediate doses of 10 μM and 25 μM ketotifen were used for experiments, with the latter used to characterize underlying signaling pathways. Decreased *ACTA2* expression observed in HDFa cells, following ketotifen treatment, was confirmed at the protein level (Fig. 1E, left and right). Reduced αSMA protein was observed in ketotifen-treated TGFβ1-activated fibroblasts compared to TGFβ1 treatment alone. While type 1 collagen (*COL1A1*) expression was not different between mock-treated and 25 μM ketotifen-treated HDFa fibroblasts at rest or following TGFβ1-activation (Fig. 1F), pro-collagen 1α1 protein levels were significantly reduced in ketotifen-treated TGFβ1-activated HDFs (Fig. 1G). Similarly, fibronectin protein levels were reduced in ketotifen-treated TGFβ1-activated HDFs (Fig. 1H). Taken together, these results suggested that ketotifen may be acting via a mechanism that inhibits the cytoskeletal rearrangement of fibroblasts towards a myofibroblast phenotype and impairs ECM deposition by HDFs.

**Figure 2:**
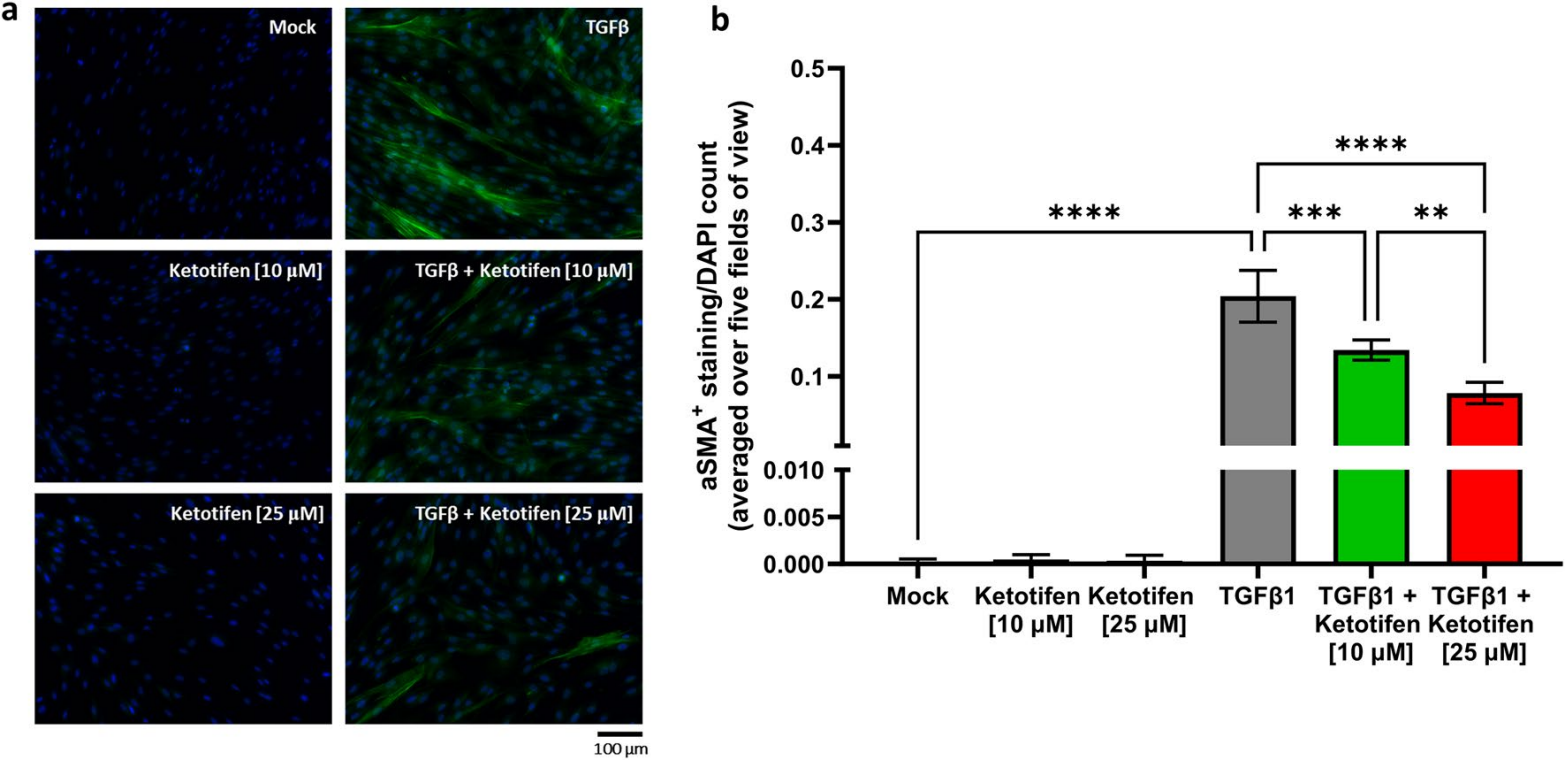
Ketotifen treatment reduced αSMA fibres in TGFβ1-activated fibroblasts. HDFa cells were cultured on poly-d-lysine-coated coverslips and treated for 48 h with media, 10 μM or 25 μM ketotifen, 10 ng/mL TGFβ1, or 10 ng/mL TGFβ1 with ketotifen added during the final 24 h. (**A**) Representative images of immunofluorescence staining (200X total magnification) probing for αSMA and DAPI are shown on the different groups of HDFa cells. (**B**) Quantification of αSMA+ staining (green) was plotted per DAPI+ cell (blue). Measurements for each biological replicate were averaged from five microscopic fields of view. Data shown as mean ± SEM. n = 3 per treatment condition. **p < 0.01; ***p < 0.001; ****p < 0.0001. αSMA, alpha-smooth muscle actin, DAPI, 4′, 6-diamidino-2-phenylindole, *HDFa* human dermal fibroblasts (adult), *SEM* standard error of the mean, *TGFβ1* transforming growth factor-beta 1.

Immunofluorescence staining demonstrated no difference in αSMA content between resting fibroblasts treated with ketotifen and mock-treated cells (Fig. 2A, left column). However, ketotifen-treated TGFβ1-activated fibroblasts revealed reduced aSMA^+^ staining, when normalized on a per cell basis, compared to TGFβ1 treatment in the absence of ketotifen (Fig. 2B). This effect was dose-dependent. The morphology of ketotifen-treated activated fibroblasts was notably more compact and spindle-shaped compared to the stellate-shaped features observed in fibroblasts treated with TGFβ1 alone (Fig. 2B, right column).

### Ketotifen reduced expression of genes involved in fibroblast contractility

**Figure 3:**
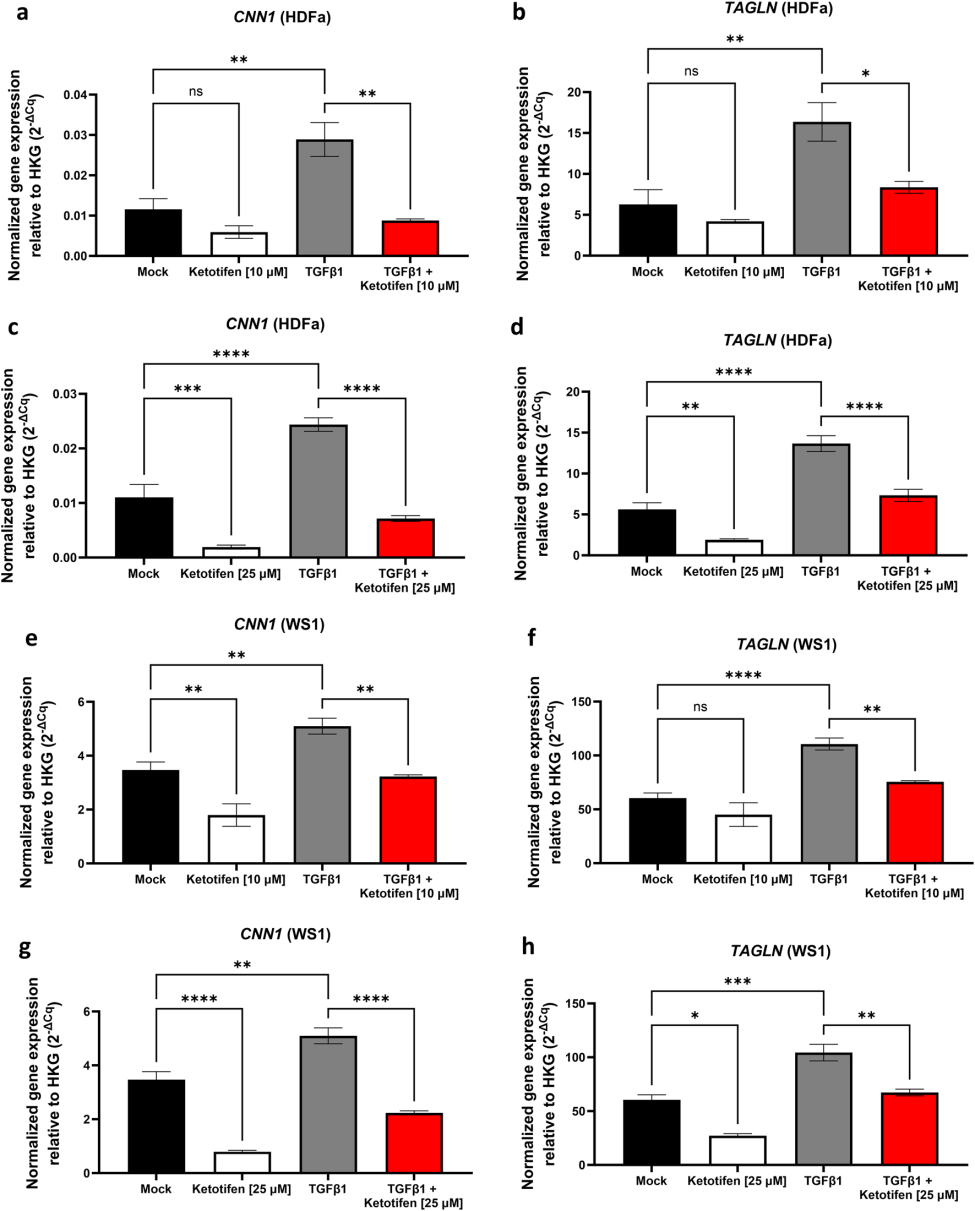
Cytoskeletal-associated genes *CNN1* and *TAGLN* are reduced with ketotifen treatment. HDFa and WS1 fibroblasts cells were treated for 48 h with DMEM supplemented with 10% FBS as mock, 10 μM or 25 μM ketotifen, 10 ng/mL TGFβ1, or 10 ng/mL TGFβ1 with ketotifen added during the final 24 h. *CNN1* and *TAGLN* expression were assessed under 10 μM ketotifen (HDFa: **A**, **B**; WS1: **E**, **F**) and 25 μM ketotifen (HDFa: **C**, **D**; WS1: **G**, **H**) conditions. Data shown as mean ± SEM. n = 3–6 per treatment condition. *p < 0.05; **p < 0.01; ***p < 0.001; ****p < 0.0001. *CNN1* calponin 1, *HDFa* human dermal fibroblasts (adult), *SEM* standard error of the mean, *TAGLN* transgelin, *TGFβ1* transforming growth factor-beta 1.

Given the impacts of ketotifen on αSMA expression, other genes involved in fibroblast morphology and contractility were assessed including actin-binding proteins calponin and transgelin (HDFa—Fig. 3A–D; WS1—Fig. 3E–H). Treatment of unstimulated fibroblasts with 10 μM ketotifen decreased gene expression of calponin 1 (*CNN1*), but only in WS1 fibroblasts (Fig. 3E). In contrast, 25 μM ketotifen treatment decreased gene expression of both *CNN1* and *TAGLN* genes in unstimulated WS1 and HDFa cells (Fig. 3C,D,G,H). TGFβ1-activated fibroblasts showed increased *CNN1* and *TAGLN*, reflecting cytoskeletal rearrangements and changes in contractility occurring as activated fibroblasts adopted a stellate-like structure. In TGFβ1-activated fibroblasts, ketotifen treatment reduced both *CNN1* and *TAGLN* expression in WS1 and HDFa cells. These findings demonstrate that transcription of several genes important in cytoskeletal rearrangements and cell contractility were impaired by ketotifen.

### YAP/TAZ axis and AKT phosphorylation are disrupted in fibroblasts with ketotifen treatment

**Figure 4:**
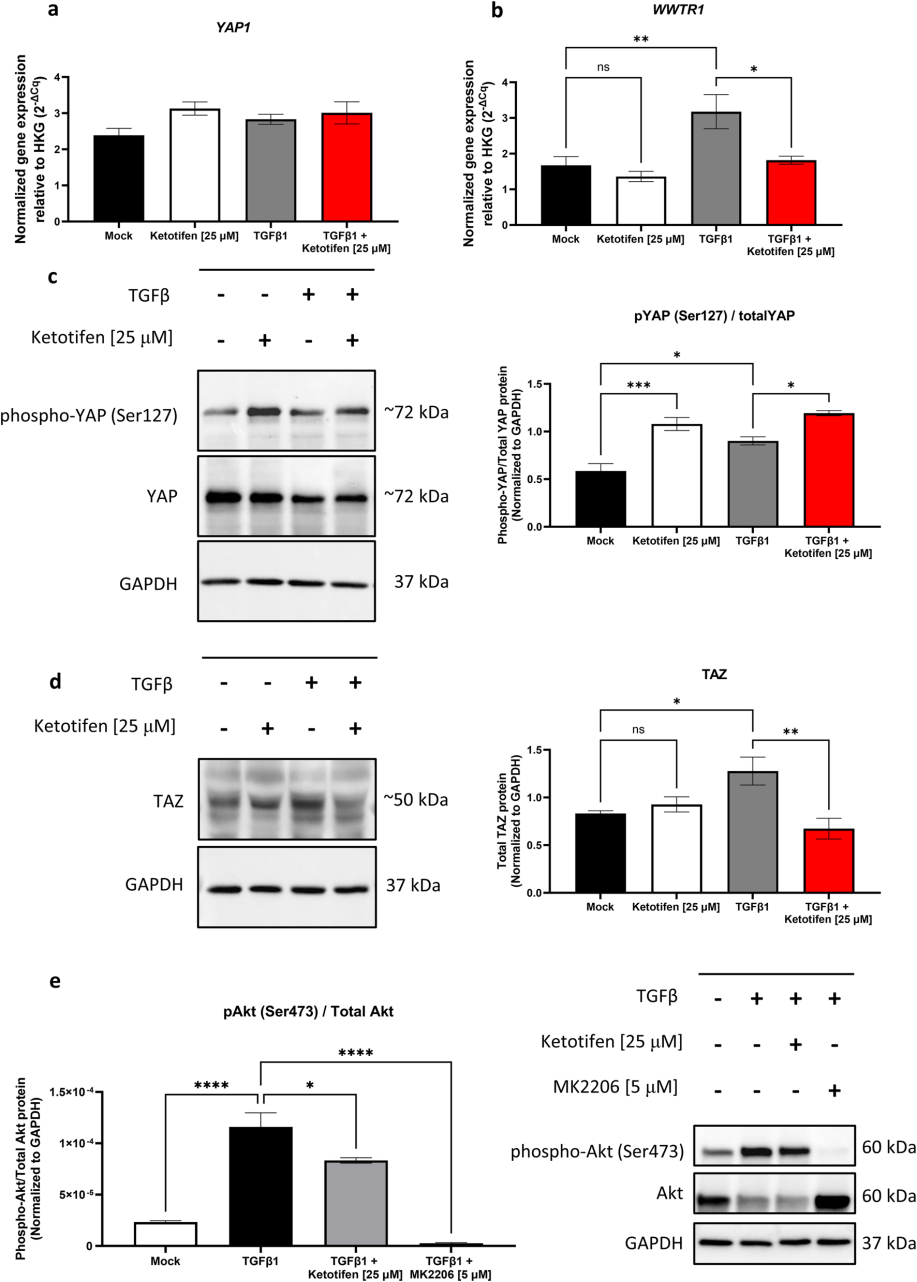
Ketotifen treatment increased phosphorylation of YAP, decreased TAZ protein levels, and inhibited AKT phosphorylation. HDFa cells were treated for 48 h with DMEM supplemented with 10% FBS as mock, 10 μM or 25 μM ketotifen, 10 ng/mL TGFβ1, or 10 ng/mL TGFβ1 with ketotifen added during the final 24 h. (**A**, **B**) Gene expression of YAP (*YAP1*) and TAZ (*WWTR1*) were assessed using RT-qPCR and normalized to housekeeping gene *HPRT*. (**C**) Phosphorylated YAP at serine residue 127 and total YAP protein were measured using western blot and semi-quantitative assessments made by normalization to GAPDH. (**D**) TAZ protein was measured and plotted in the same manner. (**E**) Representative images of the blots are shown. (**F**) Phosphorylated AKT at serine residue 473 was measured in TGFβ1-treated fibroblasts, in the presence of ketotifen or AKT inhibitor, MK2206 (left). Representative blot images of phosphorylated AKT are on the right. Full-length blots are available in Supplementary Fig. 4. Data shown as mean ± SEM. n = 3 per treatment condition. *p < 0.05; **p < 0.01; ***p < 0.001. *αSMA* alpha-smooth muscle actin, *AKT* protein kinase B, *GAPDH* glyceraldehyde 3-phosphate dehydrogenase, *HDFa* human dermal fibroblasts (adult), *SEM* standard error of the mean, *TAZ* transcriptional coactivator with PDZ binding motif, *TGFβ1* transforming growth factor-beta 1, *YAP* Yes-associated protein.

The Hippo pathway regulates the function of transcription factors YAP and TAZ, which in turn modulate cell proliferation, differentiation, and cytoskeletal responses during fibrosis^19,26,27^. Expression and phosphorylation of these molecules were examined in ketotifen-treated and untreated fibroblasts with and without TGFβ1 activation. No differences were observed in *YAP1* mRNA levels between control or TGFβ1-treated fibroblasts with or without 25 μM ketotifen treatment (Fig. 4A). However, analysis of YAP protein revealed an increase in phosphorylation at serine 127 in resting or TGFβ1-activated fibroblasts treated with ketotifen compared to controls (Fig. 4C). In addition, transcription of the gene encoding TAZ (*WWTR1*) was reduced in TGFβ1-activated fibroblasts treated with 25 μM ketotifen compared to TGFβ1 treatment alone controls (Fig. 4B), suggesting that ketotifen may also downregulate TAZ at the transcriptional level. Notably, total levels of cell-associated TAZ protein were also reduced following ketotifen treatment of TGFβ1-activated fibroblasts compared to TGFβ1-activated controls (Fig. 4D).

As protein kinase B (AKT) signaling has also been shown to be important in YAP/TAZ activation^28^, levels of phosphorylated AKT protein were determined in TGFβ1-activated fibroblasts with or without ketotifen treatment. Phosphorylation of AKT (Ser473) was impaired in TGFβ1-activated cells treated with ketotifen compared to those treated with TGFβ1 alone (Fig. 4E). To determine the role of AKT in the observed responses, the AKT inhibitor MK2206 was used to block AKT phosphorylation. TGFβ1-activated fibroblasts were treated with MK2206 (5 μM) for 24 h in the same treatment regimen as ketotifen. AKT phosphorylation at serine 473 was not observed in MK2206-treated fibroblasts (Fig. 4E). These results suggest an inhibitory function of ketotifen on signaling and transcriptional elements critical for fibroblasts to adopt a pro-fibrotic phenotype in the presence of stimuli such as TGFβ1.

### Ketotifen treatment shows greater anti-fibrotic potential after cessation of TGFβ1 treatment

Fibrosis is often a long-term process that follows from an acute tissue insult or injury. To model the longer-term effects of ketotifen treatment, a different treatment regimen was developed (Supplementary Fig. 3). Fibroblasts were initially either mock-treated or TGFβ1-treated (10 ng/mL) for 48 h. Following this, cells were washed to remove exogenous TGFβ1 and then treated with culture media (control), TGFβ1, or ketotifen for another 48 h. Notably, fibroblasts treated with ketotifen after cessation of TGFβ1 demonstrated decreased *ACTA2* and *CNN1* compared to culture media (control) cells post-TGFβ1 treatment (Supplementary Fig. 3A–C). Interestingly, no differences were seen in *WWTR1* and *COL1A1* gene transcription between ketotifen-treated and mock-treated fibroblasts post-TGFβ1 treatment. However, gene expression in both groups were substantially reduced compared to fibroblasts that continued under TGFβ1 treatment for the duration of the entire experiment (Supplementary Fig. 3E,F).

### Ketotifen treatment reduces contractility of TGFβ1-activated human fibroblasts and decreases measures of fibrosis in a murine model of dermal fibrosis

**Figure 5:**
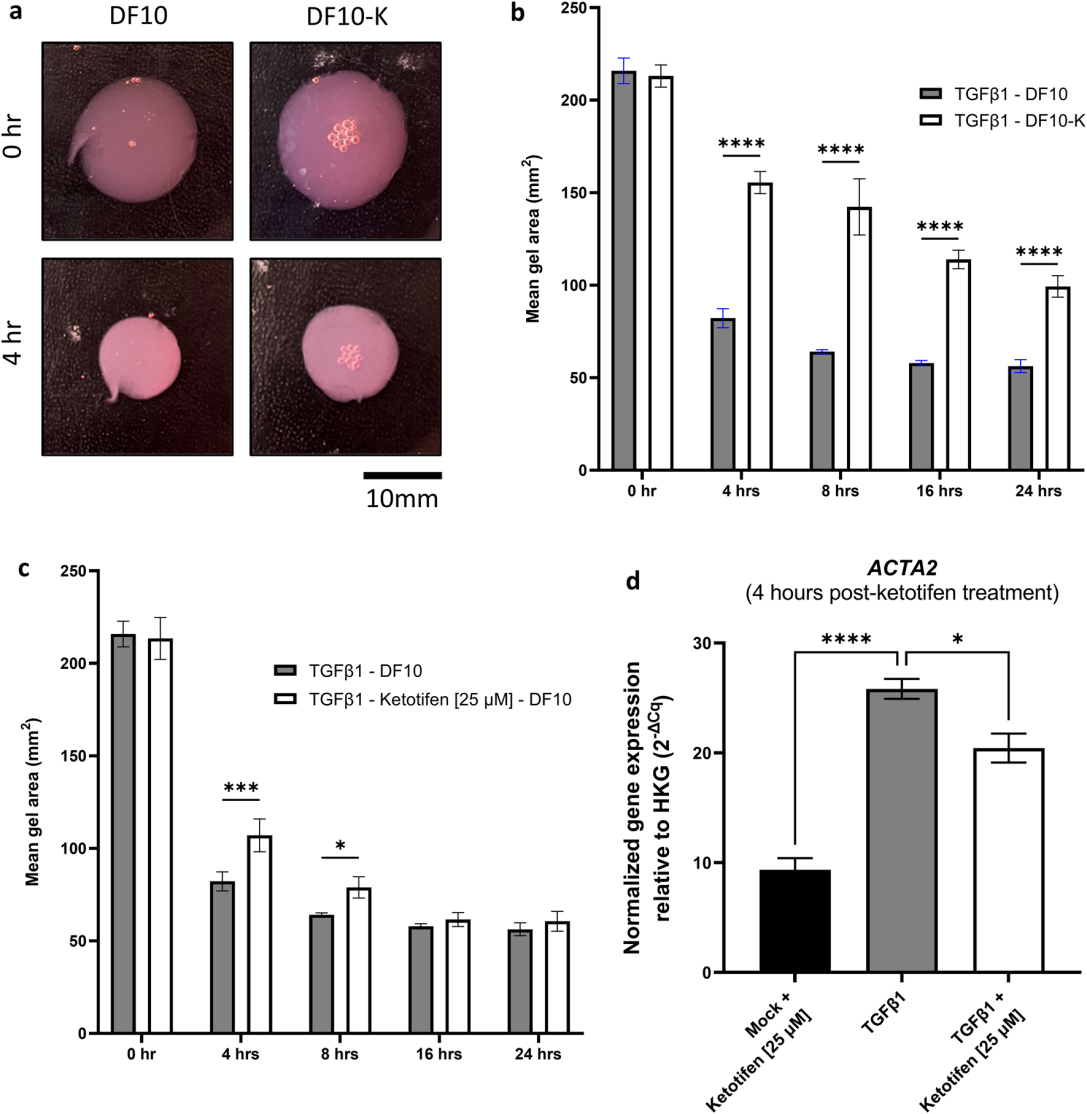
Ketotifen fumarate treatment impaired TGFβ1-stimulated fibroblast contraction in an in vitro model of fibroblast-populated gel contraction. (**A**) Representative images of fibroblast-populated collagen gels in DF10 (DMEM, with 10% FBS) culture media with or without ketotifen fumarate at 0- and 4 h post-treatment. (**B**) Contracture of activated fibroblast-populated collagen gels was determined and plotted over the course of 24 h as mean gel area in normal or ketotifen-supplemented media. (**C**) TGFβ1 or TGFβ1 and ketotifen fumarate-treated fibroblasts were seeded into collagen gels and contracture measured in normal culture media. (**D**) *ACTA2* is significantly reduced in ketotifen-treated TGFβ1-activated fibroblasts compared to TGFβ1 treatment at 4 h. Gene expression data is normalized to housekeeping gene *HPRT*. Data shown as mean ± SEM. n = 3 per treatment condition. *p < 0.05; ***p < 0.001; ****p < 0.0001. *DF10* 10% FBS-DMEM, *DF10-K* 10% FBS-DMEM with ketotifen fumarate, *TGFβ1* transforming growth factor-beta 1, *TK25* transforming growth factor-beta 1 with 25 μM ketotifen.

A key feature of pro-fibrotic myofibroblasts is their contractile ability. In an in vitro functional fibroblast-populated collagen lattice (FPCL) gel contracture assay, ketotifen diminished the contractile ability of activated fibroblasts seeded into collagen gels. As early as 4 h after gels were lifted, FPCLs in control media contracted to less than 50% of their original size, while FPCLs in ketotifen-conditioned media were around 70% of their original size (Fig. 5A,B). FPCLs continued to shrink over the next 20 h, with FPCLs in ketotifen-conditioned media demonstrating reduced contractility compared to those in normal culture media. To determine the effects of ketotifen-treatment in the context of TGFβ1-activated fibroblasts, fibroblasts activated with only TGFβ1 and seeded into FPCLs were compared with those treated with TGFβ1 and ketotifen. FPCLs with ketotifen-treated fibroblasts showed reduced contractility at 4- and 8 h compared to fibroblasts activated only with TGFβ1 (Fig. 5C).

Given the short-term impacts of ketotifen on contractility, the gene regulation at early time points following ketotifen treatment was assessed. *ACTA2* gene expression was decreased as early as 4 h after ketotifen treatment in TGFβ1-pretreated fibroblasts compared to controls without ketotifen treatment (Fig. 5D). Taken together, these results suggest that ketotifen treatment induced early transcriptional changes and influenced protein dynamics associated with contractility in TGFβ1-treated fibroblasts.

**Figure 6:**
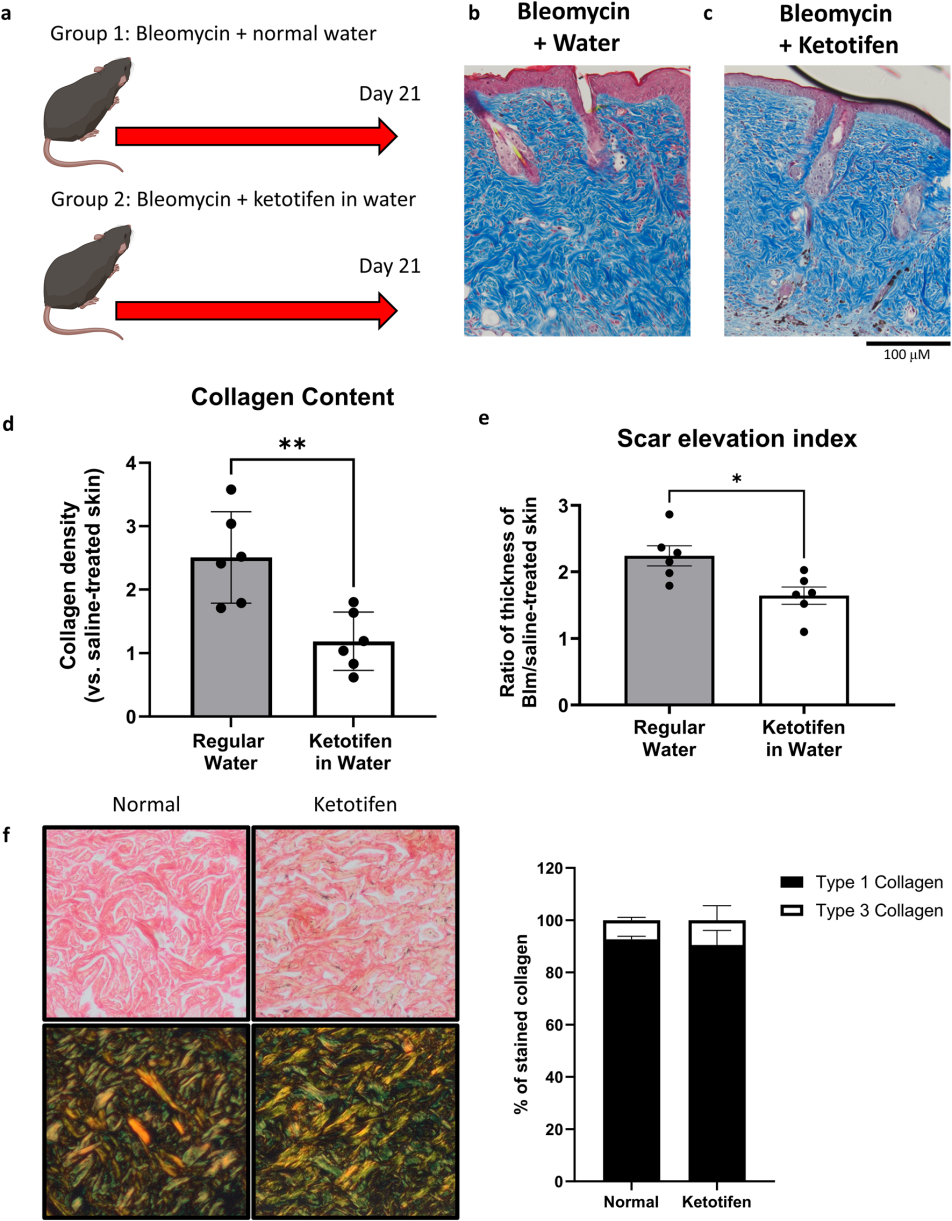
Ketotifen fumarate reduced collagen density and dermal thickness in the skin in a mouse model of bleomycin-induced skin fibrosis. Two groups of mice underwent bleomycin-induced skin fibrosis for 21 days with or without ketotifen (0.00088 mg/day) supplemented in drinking water (**A**). Representative images of bleomycin-treated skin are shown after Masson’s Trichrome histological staining for collagen, with bleomycin and regular water on the left (**B**) and bleomycin with oral ketotifen on the right (**C**). Collagen density of bleomycin-treated skin was measured, normalized to respective saline-treated skin from the same mouse, and then plotted (**D**), showing a significant decrease in ketotifen-treated fibrotic skin. Dermal thickness was measured from haematoxylin and eosin-stained skin sections and represented as scar elevation index (**E**). Representative skin sections stained with a picrosirius red staining kit are shown under brightfield microscopy (**F**, top row) and birefringence imaging (**F**, bottom row) settings. The ratio of yellow-orange and green fibres were measured as expressed as percent of stained collagen (**F**, right). Data shown as mean ± SEM. n = 5–6 per experimental group. **p < 0.01.

Since activated fibroblasts and myofibroblasts are major producers of ECM, histological measurements of dermal fibrosis were assessed in an in vivo pro-fibrotic setting in the presence or absence of ketotifen (Fig. 6A). This allowed us to determine any anti-fibrotic effects of ketotifen in a fibrotic disease setting, specifically, in a murine bleomycin-induced model of dermal fibrosis. Histological assessments of bleomycin-treated skin after three weeks of treatment, where well-established fibrosis is observed, were conducted using Masson’s trichrome staining, that stains collagen fibres blue. There was increased blue staining observed in fibrotic mouse skin (Fig. 6B) compared to fibrotic mouse skin from animals provided ketotifen (Fig. 6C). These findings suggested the presence of reduced collagen density with ketotifen treatment. Quantification of these changes in collagen density showed a significant decrease in the skin of mice given ketotifen compared to those without (Fig. 6D), suggesting an anti-fibrotic function for ketotifen in the dermal compartment. Skin sections were also stained with haematoxylin and eosin and imaged using brightfield microscopy to determine dermal thickness of bleomycin-treated skin in the presence or absence of ketotifen. These measurements are represented as Scar Elevation Index (SEI) and ketotifen-treated fibrotic skin demonstrated reduced SEI compared to without ketotifen (Fig. 6E). Picrosirius red staining of normal and ketotifen-treated fibrotic skin (Fig. 6F, top row) followed by birefringence imaging of collagen fibres (Fig. 6F, bottom row) was conducted to determine the ratio of type I and III collagen with each treatment. There were no significant differences in the ratio of these two collagen sub-types in the fibrotic skin, with or without ketotifen (Fig. 6F, right).

### Ketotifen does not alter migratory and proliferative potentials of human dermal fibroblasts

Given the effects of ketotifen on functional properties of fibroblasts such as contractility and the importance of cytoskeletal rearrangements in other functional aspects of fibroblast biology, we sought to determine if ketotifen affected migration and proliferation of HDFs. Using an in vitro scratch assay, we observed similar migratory characteristics in resting fibroblasts with or without ketotifen after scratch, as determined by percent of original scratch area remaining over time (Supplementary Fig. 4A,B). To determine the effects on fibroblast proliferation, fibroblasts were stained with a cell proliferation dye, treated with or without ketotifen, and mean fluorescent intensity of dye measured using flow cytometry 24 h later. We observed similar mean fluorescent intensity measurements between fibroblasts treated with or without ketotifen (Supplementary Fig. 5A,B), and overlaying of representative histograms corroborate these observed similarities (Supplementary Fig. 5C).

## Discussion

**Figure 7:**
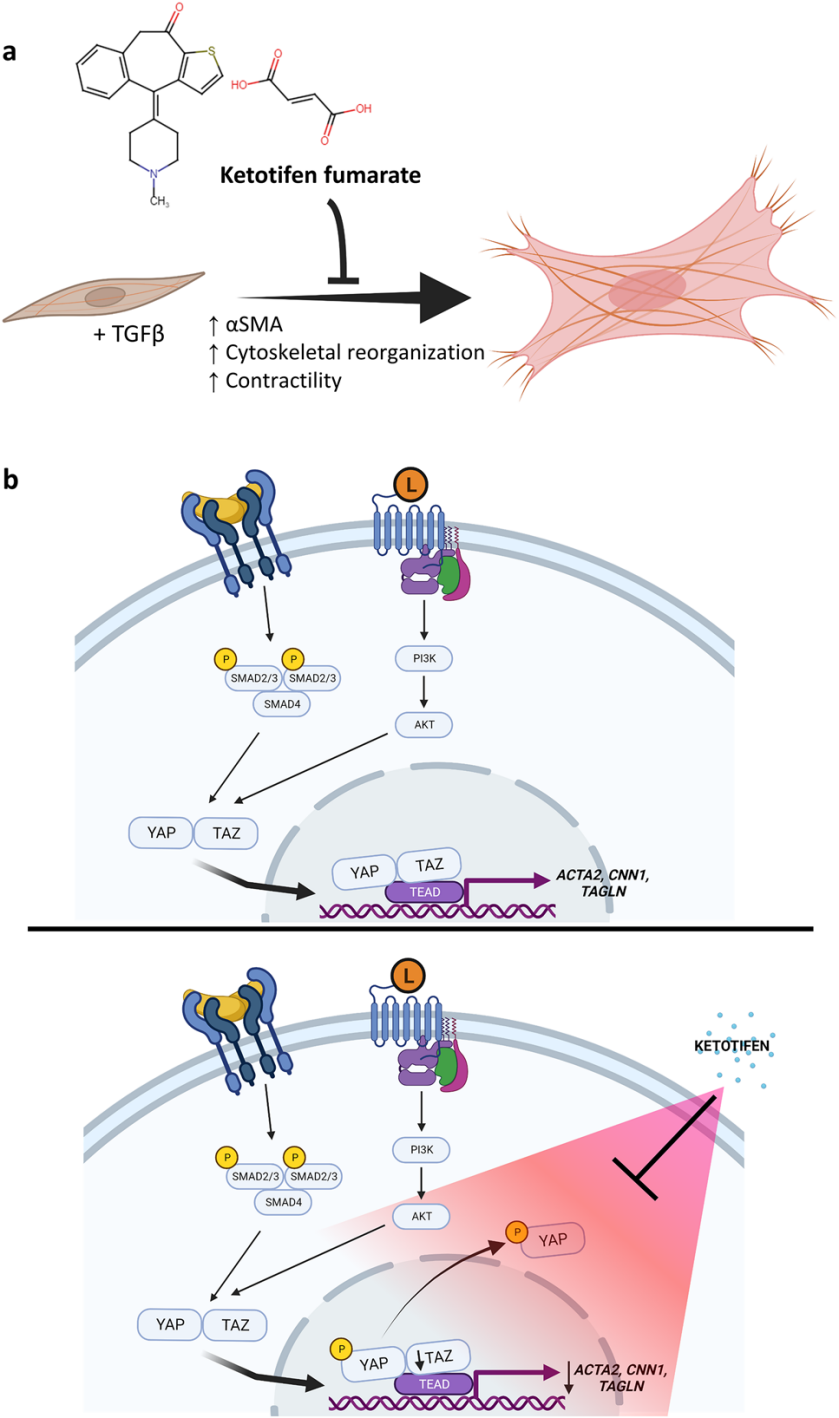
Schematic of the effects of ketotifen fumarate on fibroblast phenotype and associated cellular mechanisms. (**A**) Ketotifen fumarate inhibits the effects of TGFβ1 on fibroblasts, decreasing αSMA gene and protein levels, cytoskeletal-associated genes, and contractility. (**B**) Ketotifen fumarate treatment reduces *ACTA2*, *CNN1*, and *TAGLN*, and decreases TAZ gene and protein levels. Increased phosphorylation of YAP at serine residue 127 is also seen, promoting cytosolic localization. Ketotifen also affects the PI3K-AKT pathway by reducing phosphorylation of AKT at serine residue 473. *AKT* protein kinase B, *αSMA* alpha-smooth muscle actin, *CNN1* calponin 1, *PI3K* phosphatidylinositol-3-kinase, *TAGLN* transgelin, *TAZ* transcriptional coactivator with PDZ binding motif, *TGFβ1* transforming growth factor-beta 1, *YAP* Yes-associated protein.

In the current study, ketotifen, an antihistamine and mast cell stabilizing drug, inhibited TGFβ1-induced markers of fibrosis in human dermal fibroblasts and reduced expression of αSMA and proteins associated with cytoskeletal reorganization. Furthermore, protein levels of pro-collagen 1α1 and fibronectin were reduced by ketotifen treatment of TGFβ1-activated HDFs. Binding of TGFβ1 has been described to result in downstream effects including nuclear translocation of YAP and TAZ, which promote transcription of genes associated with cytoskeletal rearrangement and contractility^19,20^. Ketotifen administration mitigated these effects, decreasing expression of *ACTA2*, *CNN1*, and *TAGLN*. Ketotifen increased phosphorylated YAP, decreased TAZ expression and reduced the proportion of phosphorylated AKT within dermal fibroblasts. Notably, TGFβ1-stimulated fibroblasts treated with ketotifen appeared more spindle-shaped than control TGFβ1-treated fibroblasts and demonstrated decreased contractility. These changes were consistent with a ketotifen-mediated reduction in the fibrotic response to TGFβ1 stimulation, via an AKT-dependent mechanism. The effects are summarized in the schematic in Fig. 7. Finally, in an in vivo model of dermal fibrosis, ketotifen reduced dermal thickness and collagen density within fibrotic skin, suggestive of an inhibitory function towards collagen-producing fibroblasts.

Currently few targeted therapies exist to promote fibrosis resolution. The complexity of the fibrotic process involving interactions between fibroblasts, resident immune cells, and changes in the extracellular matrix adds to this challenge^29^. Currently, only two anti-fibrotic therapies have been approved for clinical use, both for treatment of idiopathic pulmonary fibrosis^30,31^. Pirfenidone targets TGFβ1-induced signaling factors leading to attenuated collagen and αSMA protein^32^, while nintedanib inhibits receptor tyrosine kinases of crucial growth factor receptors in fibrotic conditions^33^. The mast cell stabilizer ketotifen is currently used to treat allergic conjunctivitis and other allergic disorders^34,35^. In experimental models of skin wound repair and knee arthrofibrosis, ketotifen decreased fibrosis^23,24^. The noted effects of ketotifen were ascribed to the drug’s anti-inflammatory actions and stabilising activities on mast cells. We sought to determine the direct effects of ketotifen on human dermal fibroblasts, in the absence of immune cells.

TGFβ1-induced differentiation of fibroblasts is accompanied by cytoskeletal changes, most notably de novo synthesis and incorporation of αSMA^36,37^ into stress fibres^38^. This is reflected in changes in cell shape, with TGFβ1-treated fibroblasts adopting a more stellate form^39,40^. Actin fibres form adhesion structures with the ECM, allowing the cells to modulate the tissue microenvironment^41^. Due to the high tensile force of αSMA compared to other actin isoforms^42^, myofibroblasts exhibit increased contractility, which facilitates wound closure during tissue repair. Ketotifen inhibited myofibroblast formation, limiting αSMA expression and promoting retention of a spindle shape. Previous studies using an in vivo skin wound model showed that ketotifen reduced myofibroblast numbers during healing, an effect attributed to mast cell inhibition^23^. Our study demonstrates that ketotifen treatment reduces cell contractility by acting directly on fibroblasts rather than through immune effector cells, such as mast cells.

Both mechano-transduction forces from the extracellular matrix and other pro-fibrotic stimuli contribute to rearrangement and reorganization of the cytoskeleton^5,43,44^. TGFβ1-dependent activation of fibroblasts induces expression of calponins^45^ and transgelins^46^ associated with the actin cytoskeleton. These modify contractility of myofibroblasts^47–49^ and their deletion leads to reduced contractile function^49,50^. Ketotifen treatment was associated with a decrease in *CNN1* and *TAGLN* in activated fibroblasts further supporting an inhibitory role in TGFβ1-mediated cytoskeletal rearrangements and cell contractility associated with fibrosis.

The Hippo pathway effector molecules YAP and TAZ are key mediators of fibrosis^27,51–54^. These transcriptional regulators enhance *ACTA2*, *CNN1*, and *TAGLN* expression in idiopathic pulmonary fibrosis^22^. Furthermore, YAP and TAZ are upregulated upon TGFβ1 activation^55,56^, suggesting that ketotifen-induced YAP and TAZ dysregulation was responsible for decreased expression of *ACTA2*, *CNN1*, and *TAGLN*.

Phosphorylation of YAP at Ser127 promotes localization of YAP to the cytoplasm, impairing entry into the nucleus and subsequent transcription factor activity^57,58^. Increased Ser127 phosphorylation observed following ketotifen treatment provides a mechanism whereby ketotifen maintains YAP in the cytoplasm, thus inhibiting YAP-dependent transcription of pro-fibrotic factors. Ketotifen treatment of TGFβ1-activated fibroblasts also decreased TAZ protein levels reflecting decreased *WWTR1* expression. TAZ activity has been reported to drive pro-fibrotic change in fibroblasts^54,59^. Observed differences in transcript and protein levels of YAP and TAZ may partially be explained by TGFβ1 selectively promoting greater changes in TAZ than YAP^55^.

The mechanisms by which ketotifen exerts anti-inflammatory actions remain unclear. Transcriptomic evidence suggests ketotifen induced perturbations in phosphatidylinositol-3-kinase (PI3K) and protein kinase B (AKT) signaling in a Dengue virus disease model^60^. The PI3K/AKT pathway has been linked to YAP/TAZ signaling in systemic sclerosis fibrosis^61^ and AKT has been implicated in activation of YAP/TAZ in renal fibroblasts^62^. AKT phosphorylation at Ser473 is mediated by TGFβ1, eliciting pro-fibrotic downstream effects^63–65^. AKT inhibition is beneficial in animal models of pulmonary fibrosis^66^ and in mouse and human systemic sclerosis models^67,68^. Thus, the ketotifen-induced impairment of AKT phosphorylation we observed suggests a novel anti-fibrotic mechanism of action for ketotifen.

Contractility of fibroblasts and myofibroblasts is critical to wound healing and tissue repair. Ketotifen treatment reduced FPCL contracture indicative of altered fibroblast contractility. We speculate this could be due to both direct effects of ketotifen on protein components and dynamics involved in contractility, as well expression of relevant genes such as *ACTA2.* Previous reports of ketotifen-associated effects on contractility, such as reduced post-traumatic joint capsule contracture were mast cell-dependent^69^. In an in vivo rabbit model of post-traumatic joint contracture, ketotifen treatment significantly reduced contraction and reduced myofibroblasts and mast cell numbers^70^. These results were attributed to decreased mast cell activation. Our findings suggest these observations could be related to direct impacts of ketotifen. Other properties of fibroblast function associated with cytoskeletal rearrangements including migration and proliferation were not modified by ketotifen. Currently, ketotifen fumarate is in phase III clinical trials for reduction of elbow joint contracture severity (ClinicalTrials.gov ID: NCT03582176). Mast cell involvement in fibrosis remains a controversial topic. The long-established assumption that ketotifen’s effects are mast cell-dependent may not hold true. While ketotifen impairs degranulation-associated inflammation by mast cells in fibrosis, ketotifen can also mediate direct changes in fibroblast function. A primary function for fibroblasts and myofibroblasts is production of ECM such as collagen^71,72^, which was impaired by ketotifen treatment in fibrotic mouse skin as assessed by collagen density histological staining. In a similar study investigating the effects of ketotifen on a tight-skin mouse (TSK) model of dermal fibrosis, ketotifen treatment reduced fibrous band widths compared to untreated TSK mice skin^73^. Together, these results and ours suggest an anti-fibrotic effect of ketotifen in in vivo settings of skin fibrosis.

The AKT signaling axis has been linked to fibroblast contractility and mechanotransduction in multiple studies^74,75^. Furthermore, AKT inhibitors impair FPCL contraction and decrease αSMA protein levels^76^, suggesting a potential mechanism for our observed effect of ketotifen on fibroblasts. Ketotifen could be potentially beneficial in reducing dermal fibrosis particularly if localized delivery to the skin could be achieved. Moreover, the identification of the novel inhibitory activity of ketotifen on AKT could be of interest in other clinical settings. Currently, AKT inhibitors are being explored as adjuvant cancer therapies^77–79^. AKT signaling is crucial to many physiological processes including cell proliferation and apoptosis^80,81^ and therefore some AKT inhibitors have significant side effects. In contexts where partial or selective AKT inhibition is beneficial, ketotifen may provide a therapeutic option that has already demonstrated an excellent safety profile.

There are some limitations to our study, including the direct effects of ketotifen on mast cells, which cannot be disregarded in the in vivo assessments of collagen density. This is especially important considering ketotifen’s clinical used as a mast cell stabilizer. Moreover, the oral and systemic delivery of ketotifen in the mouse model may yield effects that cannot be modeled by the direct treatment of dermal fibroblasts and effects on signaling pathways modified in vitro. Future experimentation in mast cell-deficient animal models and topical local delivery systems will be beneficial in further elucidating more direct contributions and effects of ketotifen. Nevertheless, these results point to considerable therapeutic potential of ketotifen in ameliorating dermal fibrosis and warrant further investigation.

## Conclusion

These findings demonstrate that ketotifen directly reduces pro-fibrotic activities of human dermal fibroblasts, such as decreased αSMA levels, reduced ECM deposition, and impaired contractile function. These changes are associated with modified cytoskeletal protein expression and cell contractility mediated via perturbations in the YAP/TAZ and AKT signaling pathways, not previously recognised as impacts of ketotifen treatment. These direct effects of ketotifen should be considered when interpreting studies which ascribe antifibrotic effects to mast cell stabilization alone. Given that ketotifen is clinically well-tolerated, its local or systemic delivery may prove beneficial in the setting of scleroderma, burn wounds, and dermal scarring, offering novel therapeutic potential towards these debilitating skin conditions.

## Materials and methods

### Cell culture

Normal primary human dermal fibroblasts (HDFa, PCS-201-012, ATCC, Manassas, VA; isolated from skin of a 27-year-old Asian male) and normal human WS1 dermal fibroblasts (CRL-1502, ATCC; isolated from skin of a Black female at 12-weeks of gestation) were used for in vitro experiments. Fibroblasts from ATCC were obtained under informed consent, conforming to HIPAA regulations to protect privacy of the donors. HDFa cells were cultured using a low-serum media growth kit (ATCC) consisting of fibroblast basal medium supplemented with 5 ng/mL rh FGFb, 7.5 mM L-glutamine, 50 μg/mL ascorbic acid, 1 μg/mL hydrocortisone hemisuccinate, 5 μg/mL recombinant human insulin, and 2% fetal bovine serum (FBS) (Gibco, Thermo Fisher Scientific, Waltham, MA). WS1 cells were cultured in Dulbecco’s Modified Eagle Medium (DMEM, supplemented with 10% FBS. Adherent fibroblasts were detached from cell culture flasks with TrypLE Express (Gibco) and passaged when approximately 80% confluency was reached. WS1 cells were cultured in DMEM supplemented with 10% FBS. Cells were grown in humidified incubators set at 37 °C and 5% CO_2_ and passaged when approximately 80% confluent. Recombinant human transforming growth factor-β1 (TGFβ1, Peprotech, Thermo Fisher Scientific) and ketotifen fumarate (Sigma-Aldrich, St. Louis, MO) were used to treat dermal fibroblasts at 10 ng/mL and either 10 μM or 25 μM, respectively. MK2206 (Cayman Chemicals, Ann Arbor, MI) was used at 5 μM concentration. Each set of activations was repeated at least one additional time independently.

### Bleomycin-induced skin fibrosis

Male C57BL6 mice were purchased from Charles River Laboratories (Wilmington, MA) and used when 8–10 weeks of age. Two adjacent 1cm^2^ sites were shaved and depilated on the dorsal back of each mouse. In all 12 mice, intradermal injections of saline were given to one site and 50 μg of bleomycin to the other, every other day for 21 days. Six mice were randomly selected to receive regular drinking water and another six administered drinking water supplemented with ketotifen, dosed to ensure mice consume approximately 0.00088 mg per day (equivalent to recommended dose for humans). Food and water were provided ad libitum and from the same source. Cages were maintained adjacent to each other in the same conditions to negate any environmental variations. After 21 days, mice were euthanized (anesthetization with isoflurane until a lack of pedal reflex [an indicator of pain], asphyxiation with CO_2_, and cervical dislocation to further ensure death), and skin tissues taken for blinded histological assessment. All animal studies were approved by the University Committee on Laboratory Animals at Dalhousie University and all experiments were conducted in accordance with relevant guidelines and regulations.

### Real-time polymerase chain reaction (PCR)

Cells were lysed in PureZOL RNA isolation reagent (Bio-Rad Laboratories, Hercules, CA) and combined with chloroform prior to centrifugation at 12,000×*g* for 15 min. The aqueous phase was separated and mixed with equal volume of 70% ethanol, transferred to RNA purification columns, and processed using a RNeasy kit following manufacturer’s protocol (Qiagen, Hilden, Germany). cDNA was synthesized using a QuantiTect Reverse Transcription Kit (Qiagen) following manufacturer’s protocol and then used for gene expression. Gene expression analyses were conducted using quantitative real-time PCR on a Bio-Rad CFX96 RT-PCR system (Bio-Rad Laboratories) and corresponding CFX Maestro v1.1 software. Intron-spanning primers were purchased from Bio-Rad or Qiagen, or custom-designed using SnapGene software and purchased from Integrated DNA Technologies (Coralville, Iowa). Sequences for custom-designed primers are as follows: *CNN1*—forward: 5′-CTGGCTGCAGCTTATTGATG-3′, reverse: 5′-CTGAGAGAGTGGATCGAGGG-3′, *TAGLN*—forward: 5′-CTCATGCCATAGGAAGGACC-3′, reverse: 5′-TCCGAACCCAGACACAAGT-3′, *YAP1*—forward: 5′-TGTCCCAGATGAACGTCACAGC-3′, reverse: 5′-TGGTGGCTGTTTCACTGGAGCA-3′, *WWTR1*—forward: 5′-GAGGACTTCCTCAGCAATGTGG-3′, reverse: 5′-CGTTTGTTCCTGGAAGACAGTC-3′.

### Western blot and ELIA analysis

Fibroblasts were lysed in 1X Radio-Immunoprecipitation Assay buffer (Sigma-Aldrich) with the addition of 1X PhosSTOP (Roche, Sigma-Aldrich) phosphatase inhibitor and 1X cOmplete Mini (Roche, Sigma-Aldrich) protease inhibitor. Protein concentrations were determined from cleared lysates using Pierce BCA Protein Assay Kit (Thermo Fisher Scientific) and 30 μg of protein taken for electrophoresis in 12–15% sodium dodecyl sulfate–polyacrylamide gels or Mini-PROTEAN precast gels (Bio-Rad Laboratories). Gels were transferred to nitrocellulose membranes, blocked with either 5% non-fat milk or bovine serum albumin (BSA), probed with primary and corresponding secondary antibodies, detected with Clarity Max Western ECL Substrates (Bio-Rad Laboratories), and imaged and processed using a ChemiDoc Imaging System and corresponding ImageLab software (Bio-Rad Laboratories). Exposures were automatically determined using the ChemiDoc Imaging System. Densitometric analyses were performed using ImageJ software (version 1.54f., National Institutes of Health, Bethesda, MD). To quantify protein levels released by fibroblasts, pro-collagen 1α1 and fibronectin, supernatants were collected at experimental endpoints and ELISAs were conducted using kits from R&D Systems (Minneapolis, MN), following manufacturer’s protocol.

### Immunofluorescence

Fibroblasts seeded and activated on poly-d-lysine-coated (Sigma-Aldrich) coverslips were permeabilized and fixed with a 95% ethanol-5% glacial acetic acid solution and washed several times with phosphate-buffered saline (PBS, Gibco). Coverslips were blocked with 10% goat serum, incubated with primary and corresponding secondary antibodies, incubated with 0.5 μM DAPI, and mounted onto microscope slides. Slides were imaged under a Cy3 filter using the Mantra Quantitative Pathology Imaging System and processed using corresponding inForm Automated Image Analysis software (Akoya Biosciences, Marlborough, MA). Measurements were taken across five fields of view and averaged. DAPI + cells were identified and quantified using inForm software. αSMA staining was quantified using ImageJ software.

### Histological assessments of dermal fibrosis

To assess collagen density, 5 μm thick formalin-fixed and paraffin-embedded murine skin sections were taken for Masson’s trichrome staining. Slides containing skin sections were rehydrated through a series of xylene and ethanol washes, followed by an overnight incubation in Bouin’s solution (Sigma-Aldrich). The next day, Bouin’s solution was rinsed away using cold tap water and slides immersed in Weigert’s working haemotoxylin (Sigma-Aldrich) for 5 min, followed by another 5 min wash under cold running tap water. Slides were then stained using a Masson’s Trichrome Staining Kit (Abcam, Cambridge, United Kingdom) following manufacturer’s protocol, followed by dehydration through ethanol and clearance in xylene. Slides were then mounted with Epredia Cytoseal Mountant 60 (Fisher Scientific) and allowed to air-dry overnight. To assess scar elevation index, deparaffinized and rehydrated skin sections were stained with Harris’ Haematoxylin (Sigma-Aldrich) for 3 min, followed by rinsing in tap water for 3 min. Slides were then differentiated by immersion into a 1% acid alcohol solution twice for 5 s each, followed by a quick rinse in tap water. Slides were incubated in Scott’s Tap Water solution (3.5 g sodium bicarbonate, 20 g magnesium sulfate, and 1L tap water) for a minute to turn nuclei blue, followed by an immediate immersion into running tap water. Slides were rinsed in 70% ethanol followed by two dips into an Eosin solution (Leica Biosystems, Wetzlar, Germany) for 5 s each. Sections were dehydrated through ethanol, cleared in xylene, and mounted as above. Dermal thickness of bleomycin-treated skin was determined by measuring the distance between the epidermal-dermal border and dermal-hypodermal border. Images were taken using brightfield microscopy on an Olympus BX43 microscope (Leica Microsystems), and analyzed using ImageJ software, utilizing a Masson’s trichrome assessment plug-in and established protocol^82^. To determine collagen type I and type III ratios, skin sections were rehydrated as above and stained using a Picro Sirius red connective tissue staining kit (Abcam). Slides were sent to the Department of Pathology at the Izaak Walton Killam Health Centre, for birefringence imaging. Birefringent images were analyzed using ImageJ software, using a published and publicly available birefringence image analysis plugin (https://github.com/TCox-Lab/PicRed_Biref). Collagen density and dermal thickness of bleomycin-treated skin was first normalized to respective saline-treated skin from the same mouse before being plotted. Each skin specimen was given a slide number that was revealed after collagen density and dermal thickness calculated to ensure blinded assessments were maintained.

### Flow cytometry

Activated fibroblasts were processed using an Annexin V Apoptosis Detection kit (eBioscience, Thermo Fisher Scientific) following manufacturer’s guidelines. 7-Aminoactinomycin D (Thermo Fisher Scientific) was added to samples 5 min prior to acquisition using BD FACSCanto II and FACSDIVA software, and analyzed using FlowJo software (BD Biosciences, San Jose, CA). Resting fibroblasts were incubated with cell proliferation dye eFluor 670 (eBioscience) following manufacturer’s guidelines, prior to incubation with culture media in the presence or absence of ketotifen. After 24 h, fibroblasts were fixed with 1% paraformaldehyde and taken for flow cytometry acquisition using BD FACSCelesta and FACSDIVA software, and analyzed using FlowJo software (BD Biosciences).

### Fibroblast-populated collagen gel contraction assay

HDFs were untreated or treated for 48 h, and seeded into collagen gels prepared from soluble rat tail collagen (Advanced BioMatrix, San Diego, CA) as previously described^83,84^. Gels were released from the dish and imaged over 24 h. Gel area was measured and analyzed using ImageJ software.

### In vitro scratch-wound assay

HDFs were seeded into a monolayer and allowed to adhere overnight. When 80% confluency was reached, cell culture media was refreshed, with or without ketotifen. Immediately following media replacement, the tip of a sterile 1000 μL pipette was used to scratch a vertical line down the center of the plastic culture surface, generating a gap in the fibroblast monolayer. Images of the fibroblast and scratch were taken using brightfield microscopy immediately following gap generation, and after 6, 12, and 24 h, and processed using ImageJ software to determine percent of original scratch area remaining. Markings were created on the underside of tissue culture surface to ensure the same area was imaged at each time point.

### Antibodies

Primary antibodies for western blotting and immunofluorescence were anti-AKT (#9272; Cell Signaling Technology, Danvers, MA), anti-αSMA (1A4, Abcam), anti-phospho-AKT (Ser473; D9E, Cell Signaling Technology), anti-phospho-YAP (Ser127; D9W2I, Cell Signaling Technology), anti-YAP (D8H1X, Cell Signaling Technology), anti-TAZ (1M19, Sigma-Aldrich), and anti-GAPDH (D4C6R, Cell Signaling Technology). Primary antibody concentrations used were as recommended for the assay by the manufacturer. Secondary antibodies for western blotting were mouse anti-rabbit (sc-2357, Santa Cruz Biotechnology, Dallas, TX) and donkey anti-mouse (715-035-151, Jackson ImmunoResearch Laboratories, West Grove, PA) and used at 1:2000 dilution. Secondary antibody for immunofluorescence was Alexa Fluor 546-conjugated goat anti-mouse (A11030, Invitrogen, Thermo Fisher Scientific) and used at 1:500 dilution. Antibodies used for protein concentration measurements were from pro-collagen 1α1 DuoSet ELISA (DY6220-05, R&D Systems) and fibronectin DuoSet ELISA (DY1918-05, R&D Systems) staining kits.

### Statistical analyses and graphing

Student’s t-test or one-way analysis of variance (ANOVA) were used to perform statistical analyses where appropriate, using Šidák correction for multiple comparisons. Statistical significance was set at *p* < 0.05. Graphs were generated using GraphPad Prism (version 9.5.1, GraphPad Software, San Diego, CA).

### Ethical approval

All procedures involving animals were approved by the University Committee On Lab Animals committee for use in the Carleton Campus Animal Facility at Dalhousie University. Where applicable, the study was conducted and reported in accordance with the ARRIVE 2.0 guidelines.

### Supplementary Information

Supplementary Information.

## Data Availability

The data analyzed for this study are available upon reasonable request to the corresponding authors. All data generated and analyzed are included in this article and Supplementary Materials. No datasets were generated or analyzed during the current study.
