# Supplementary material for: Ketotifen directly modifies the fibrotic response of human skin fibroblasts
Source: Sci Rep. 2024 Mar 25;14:7076. doi: 10.1038/s41598-024-57776-7 (PMC10963735; doi:10.1038/s41598-024-57776-7)

## SUPPLEMENTARY FIGURE CAPTIONS

**Supplementary Figure 1: *ACTA2* decreased in TGFβ1-activated fibroblasts with increasing ketotifen concentrations.** Gene expression of αSMA (*ACTA2*) was determined by RT-qPCR in TGFβ1-stimulated human dermal fibroblasts treated with various doses of ketotifen fumarate and normalized to housekeeping genes. Data shown as mean ± SEM. n = 3 per treatment condition. ****p*<0.001; *****p*<0.0001. αSMA, alpha-smooth muscle actin; TGFβ1, transforming growth factor-beta 1.

**Supplementary Figure 2: Ketotifen fumarate treatment did not induce significant changes in cell death in human dermal fibroblasts.** Representative flow cytometric plots are shown with percentages from fibroblasts stained for Annexin-V and 7-aminoactinomycin D, under different activation conditions (7AAD, 7-aminoactinomycin D; AnnV, Annexin-V; TGFβ1, transforming growth factor-beta 1.

**Supplementary Figure 3: *ACTA2, CNN1, TAGLN, WWTR1,* and *COL1A1* but not *YAP1* are reduced in post-TGFβ1-treated HDFa cells, after replacement with culture media with or without ketotifen.** Gene expression patterns of *ACTA2* (A), *CNN1* (B), *TAGLN* (C)*, YAP1* (D), *WWTR1* (E), and *COL1A1* (F) in HDFa cells were assessed by RT-qPCR after 96 hours under various treatment conditions. Treatment and media were replaced daily. Gene expression is normalized to housekeeping gene *HPRT*. Data shown as mean ± SEM. n = 3-6 per treatment condition. *p<0.05; **p<0.01; ***p<0.001; ****p<0.0001. HDFa, human dermal fibroblasts (adult); SEM, standard error of the mean; TGFβ1, transforming growth factor-beta 1.

**Supplementary Figure 4: Ketotifen did not affect dermal fibroblast migration in a scratch-wound migration assay.**

Representative images of scratch area were taken of wells containing HDFs in serum-free media with or without ketotifen (A). Yellow dotted lines show the original scratch area imaged immediately after gap generation. Percent of scratch area was determined using ImageJ area analysis and plotted (B). Data shown as mean ± SEM. n = 3 per treatment condition.**Supplementary Figure 5: Ketotifen did not affect proliferation of dermal fibroblasts.**

HDFs were stained using a cell proliferation dye (eFluor670) and incubated with or without ketotifen in culture media. Staining intensity was determined by flow cytometric analysis after 24 hours for each treatment condition. Representative histograms for each treatment condition are shown (A, B). Histograms were superimposed to compare peaks and mean fluorescent intensity as determined by flow cytometric analysis software (C).

**Supplementary Figure 6: Western blot images with superimposed ladders.**

Full length representative western blot membranes are shown, with probed target protein superimposed onto colorimetric images of protein ladders. Numbers on ladder represent known kilodaltons (kDa) flanking protein of interest. (A, left) αSMA observed band size is approximately 42 kDa, and membrane was stripped and re-probed for GAPDH (A, right) with observed band size of approximately 36 kDa. Total TAZ protein (B, left) was observed around 49 kDa and re-probing the membrane for GAPDH (B, right) showed a band size of approximately 36 kDa. Phosphorylated YAP at serine 127 (C, left) was observed between 65 and 78 kDa as stated by the manufacturer, stripped and re-probed total YAP (C, centre) was observed between 65 and 78 kDA as per manufacturer's datasheet, and re-probing the same membrane for GAPDH (C, right) showed an observed band size of around 36 kDa. The membrane to probe for phosphorylated AKT at serine 473 (D left) was cut at 50 kDa to allow for concurrent probing for

GAPDH (D, right). Observed band sizes were around 60 kDa and 36 kDa, respectively. The membrane probed for phosphorylated AKT at serine 473 was stripped and re-probed for total AKT (D, middle), with an observed band size of around 60 kDa.

**Supplementary Figure 7: Full western blot images with treatment groups annotated.**

Full images are shown of blots used for representative western blot images in Figure 1E and 4E (left image), with treatment groups annotated on corresponding lanes. Other lanes are either biological replicates or samples tested for unrelated experiments on the same gel. Images of the western blot membranes display the following: (A) αSMA, (B) αSMA superimposed on protein ladder, (C) GAPDH loading control probed on membrane used for αSMA staining, (D) phosphorylated YAP, (E) total YAP probed on membrane used for phosphorylated YAP staining, and (F) GAPDH loading control probed on membrane used for phosphorylated YAP and total YAP staining.

**Supplementary Figure 8: Full western blot images without annotations.**

Full and original images are shown of blots used for representative western blot images in Figure 4E (right image) and 4F, without annotations. Images of the western blot membranes display the following: (A) Total TAZ, (B) GAPDH loading control probed on membrane used for total TAZ staining, (C) phosphorylated AKT, (D) total AKT probed on membrane used for phosphorylated AKT staining, and (E) GAPDH loading control probed on divided bottom half of membrane used for phosphorylated AKT and total AKT staining.

Supplementary Figure 1: *ACTA2* decreased in TGFβ1-activated fibroblasts with increasing ketotifen concentrations.

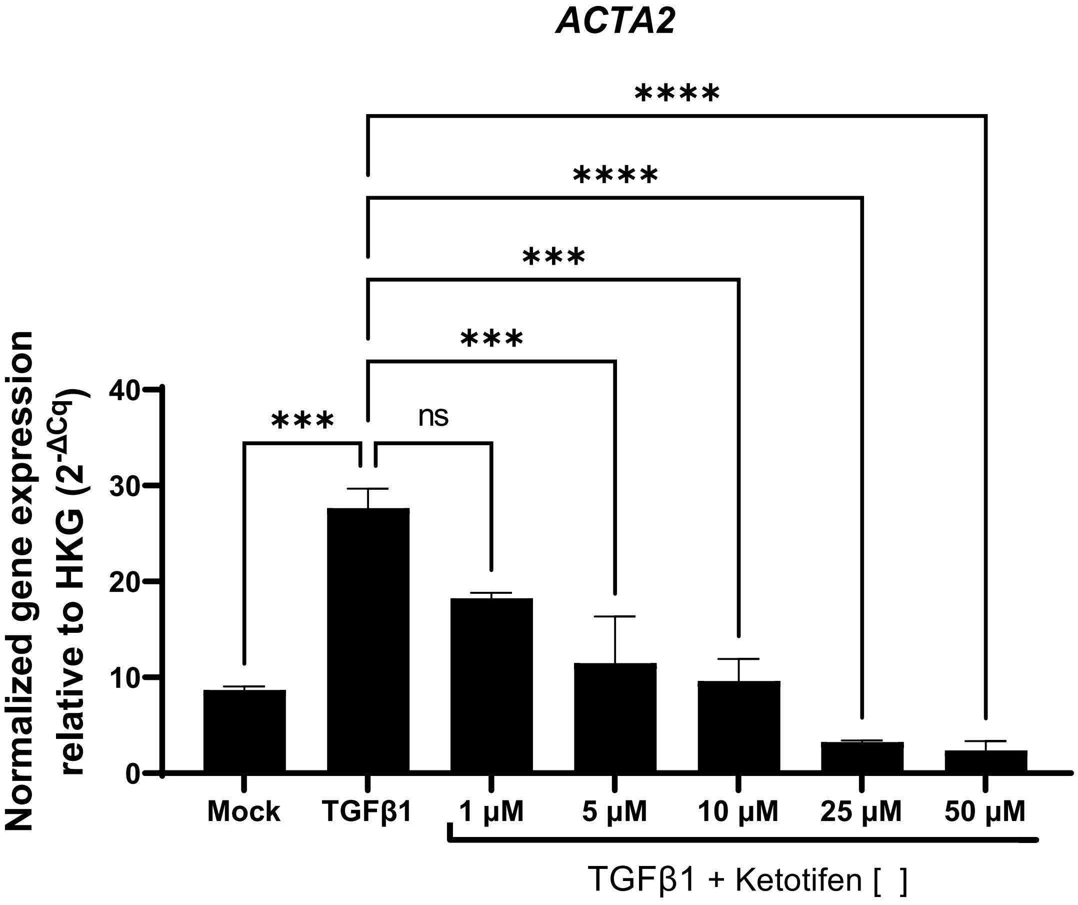

Supplementary Figure 2: Ketotifen fumarate treatment did not induce significant changes in cell death in human dermal fibroblasts.

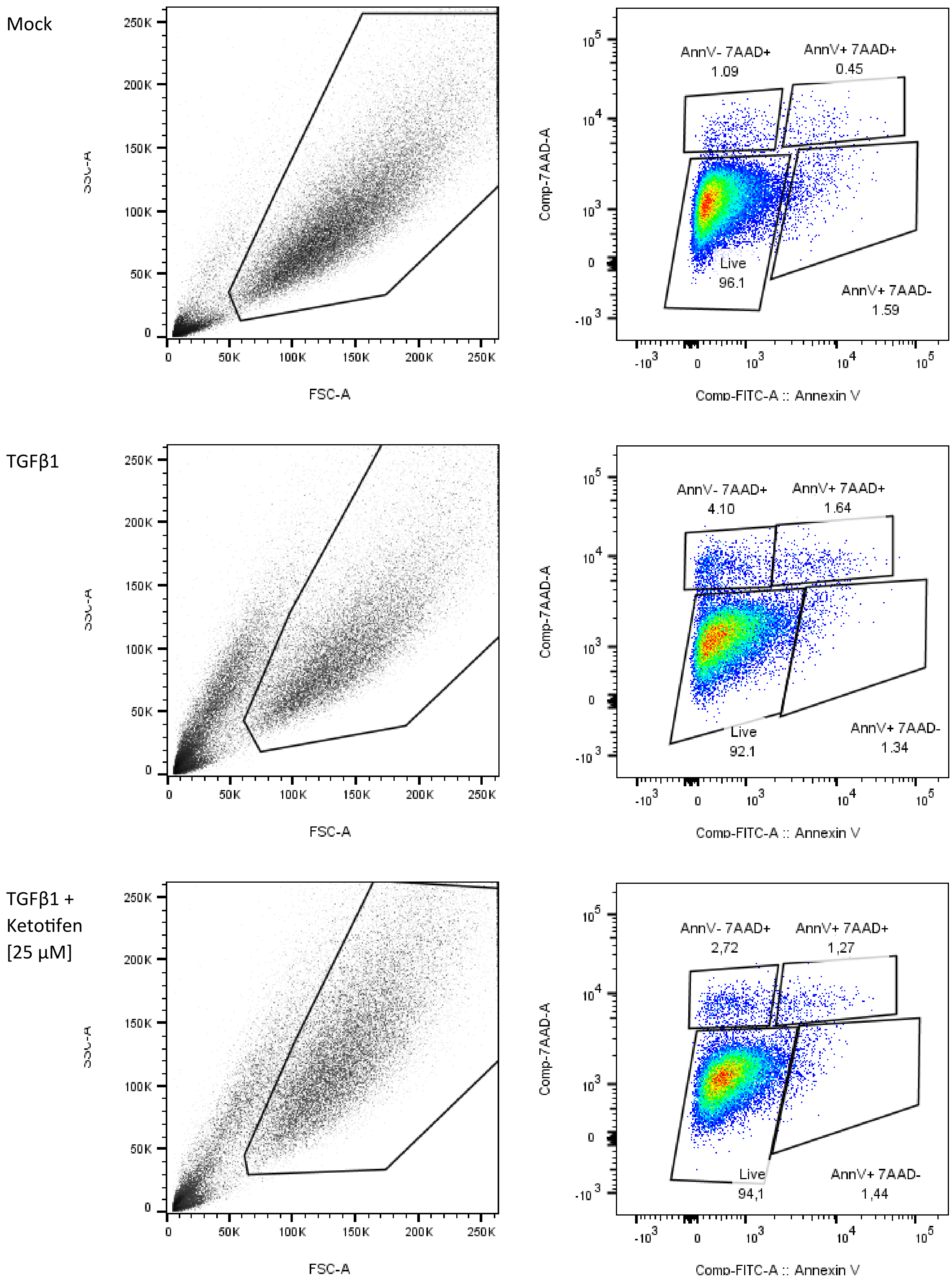

Supplementary Figure 3: *ACTA2*, *CNN1*, *TAGLN*, *WWTR1*, and *COL1A1* but not *YAP1* are reduced in post-TGFβ1-treated HDFa cells, after replacement with culture media with or without ketotifen.

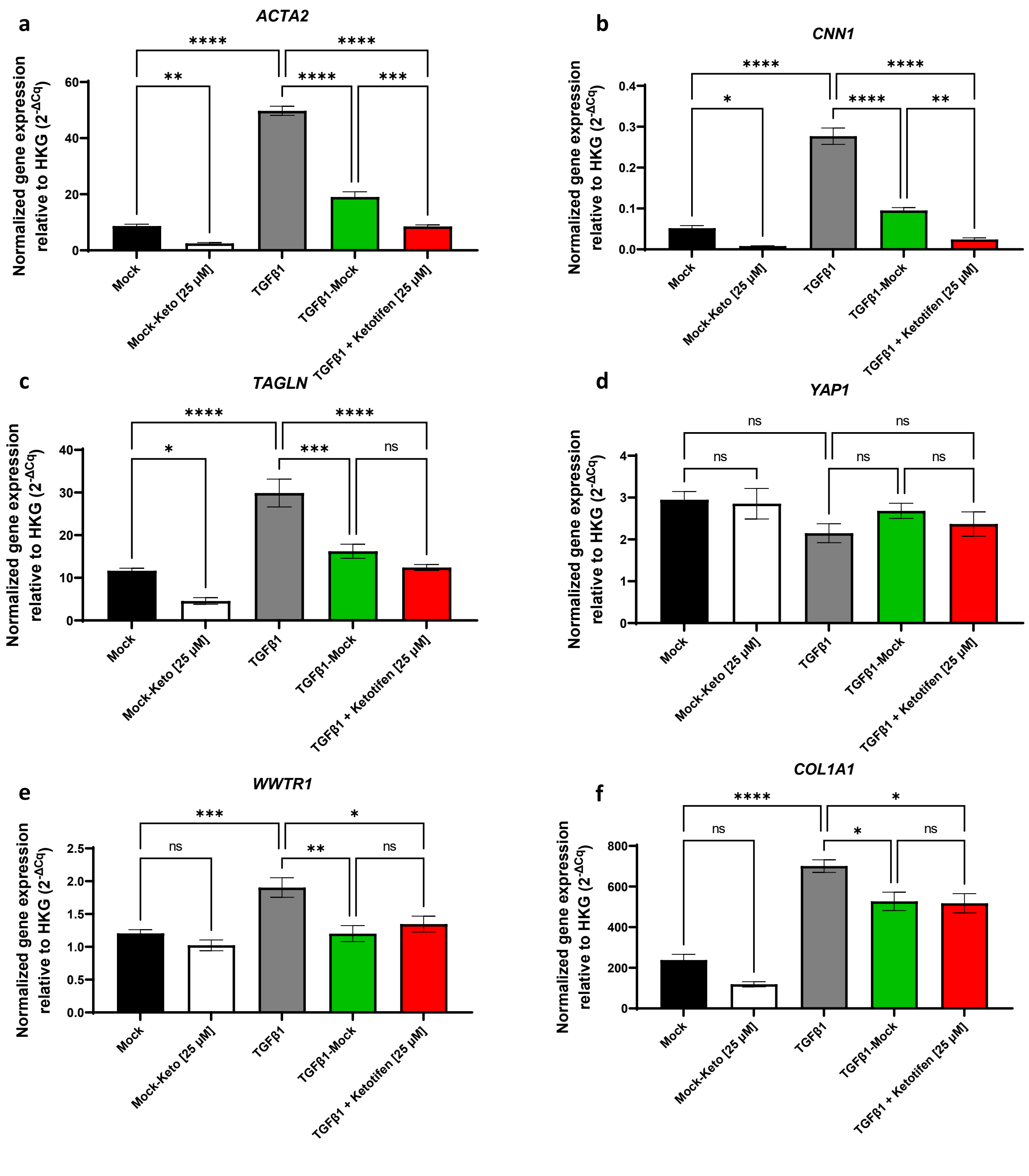

Supplementary Figure 4: Ketotifen did not affect HDF migration in a scratch-wound migration assay.

**a**

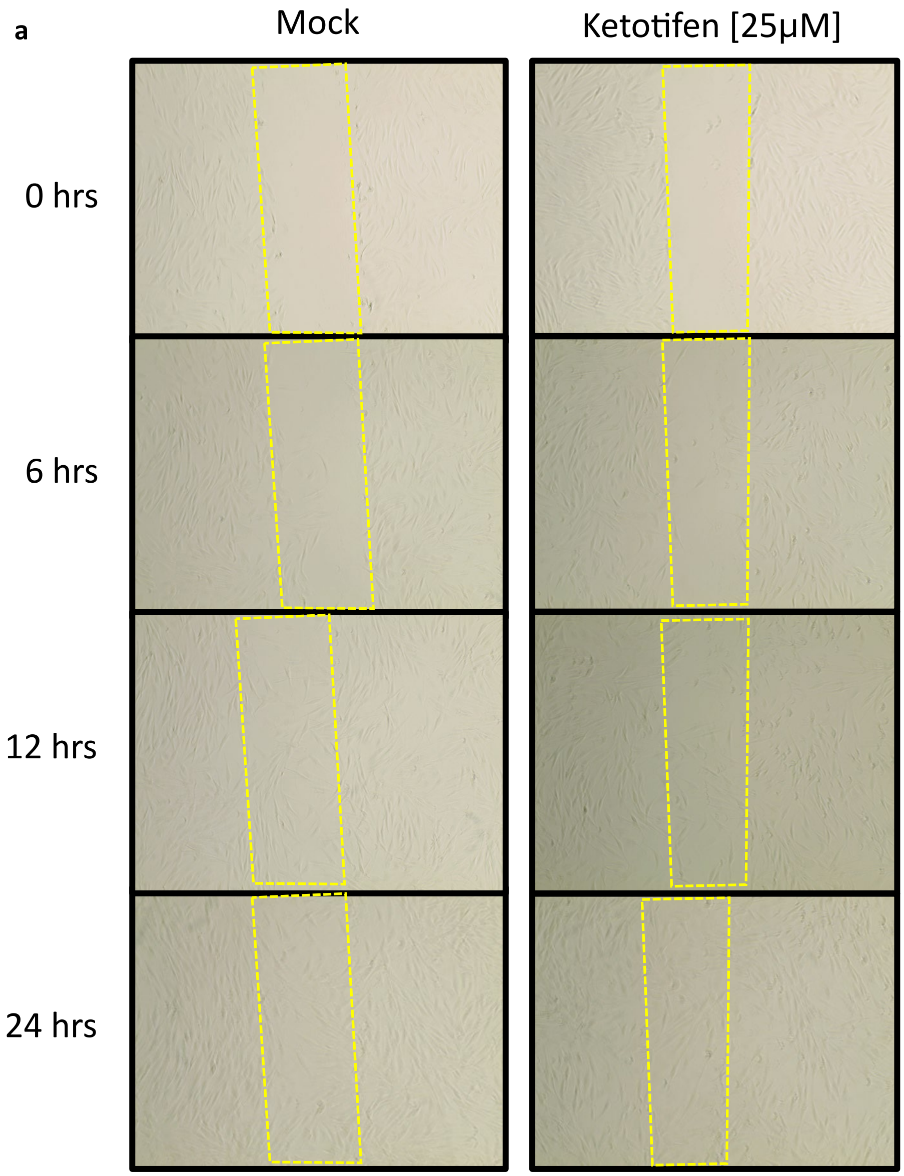


**b**

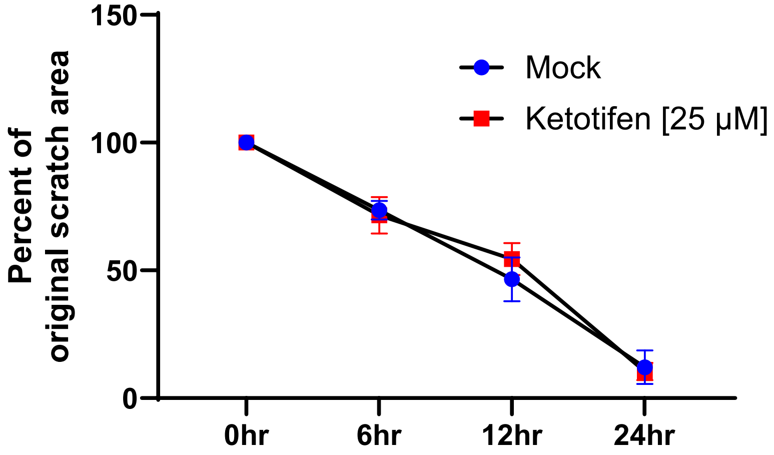

Supplementary Figure 5: Ketotifen did not affect proliferation of dermal fibroblasts.

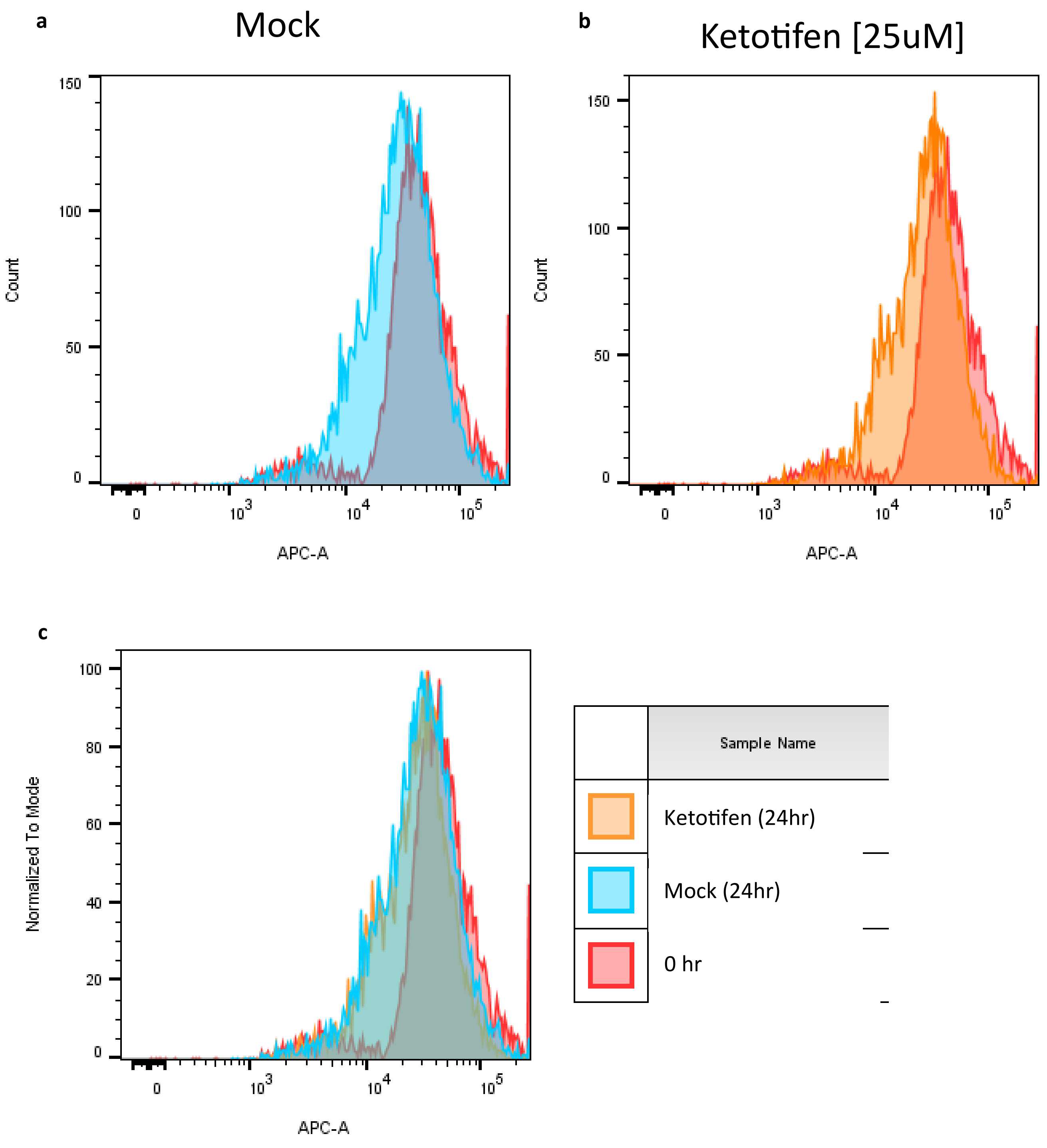

Supplementary Figure 6: Western blot images with superimposed ladders

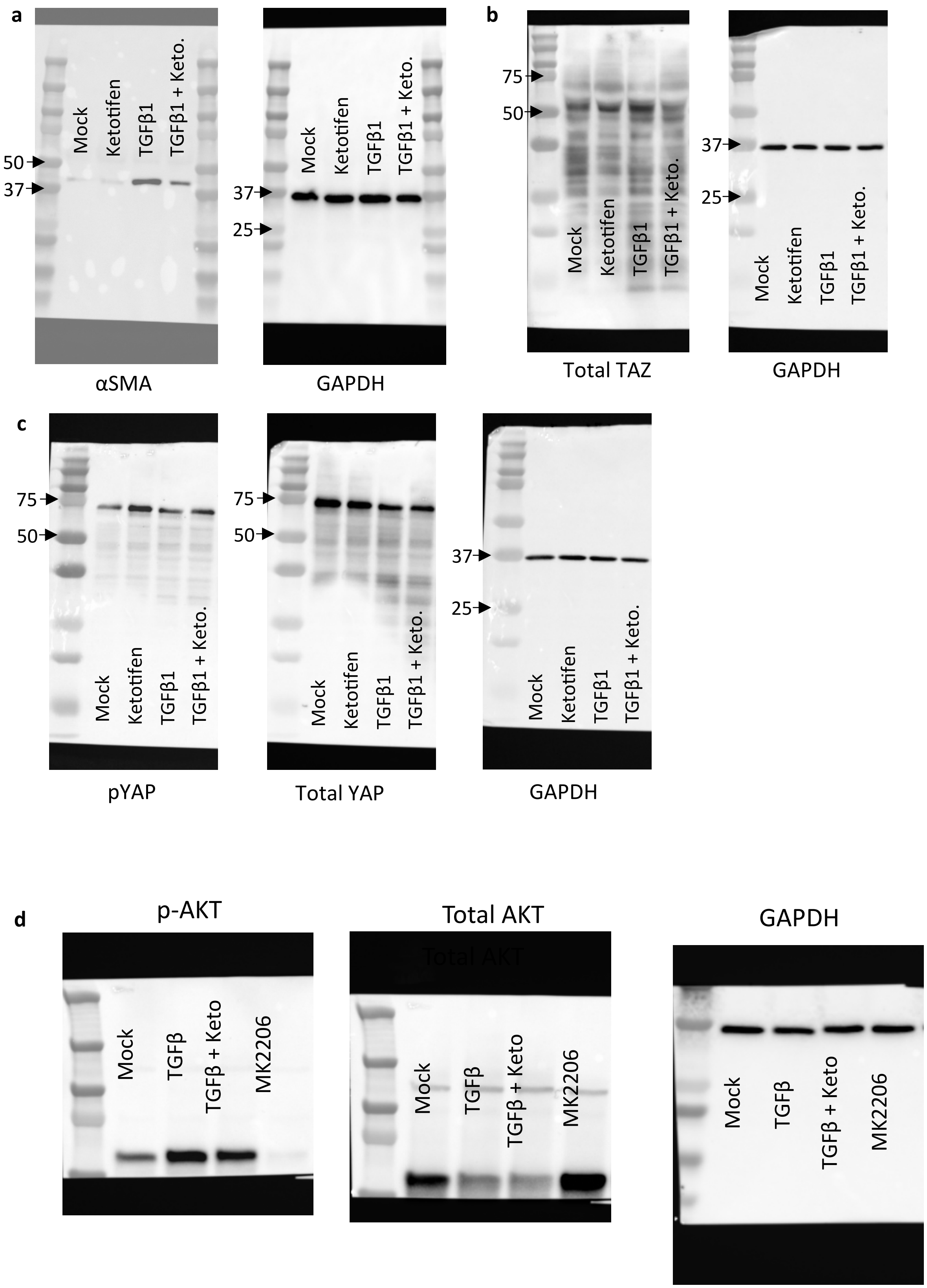

Supplementary Figure 7: Full western blot images with treatment groups annotated

**a**

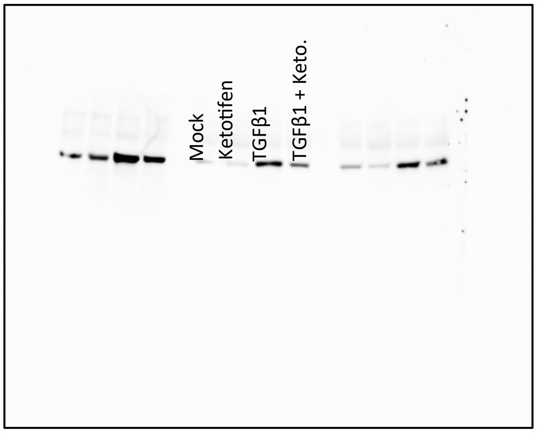


**b**

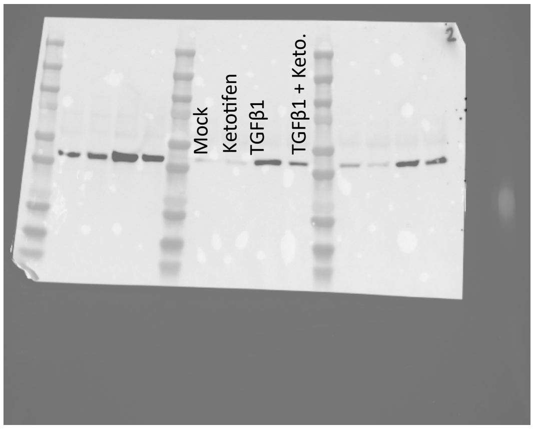


**c**

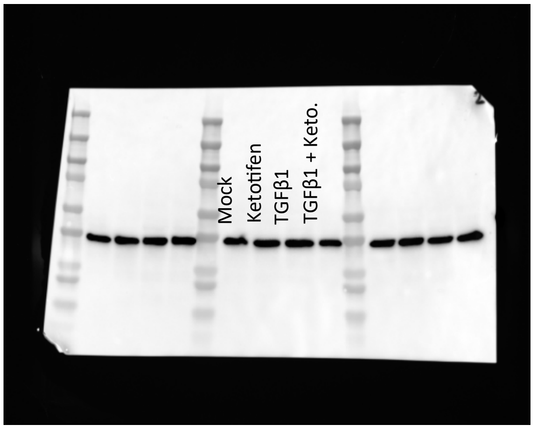


**d**

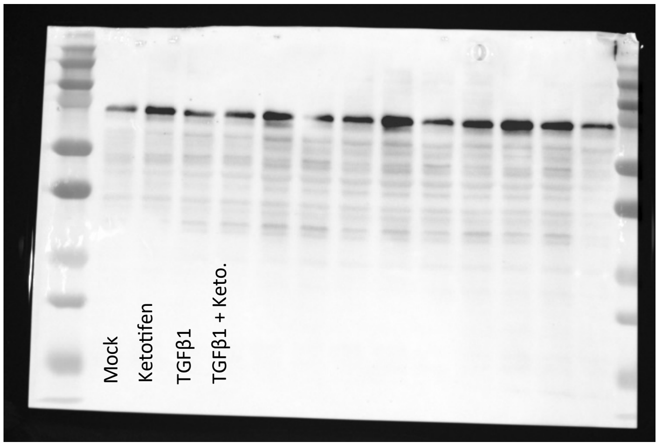


**e**

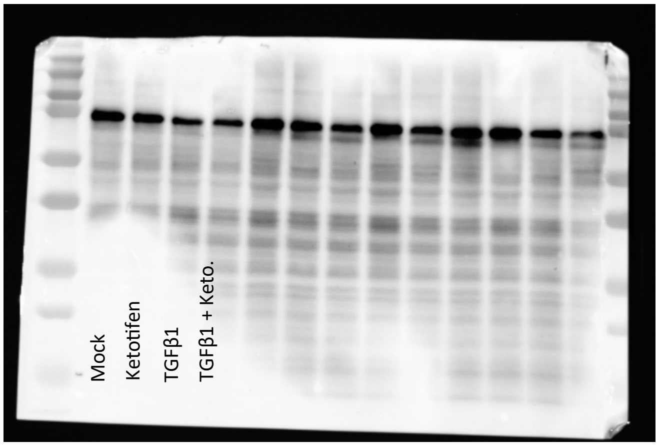


**f**

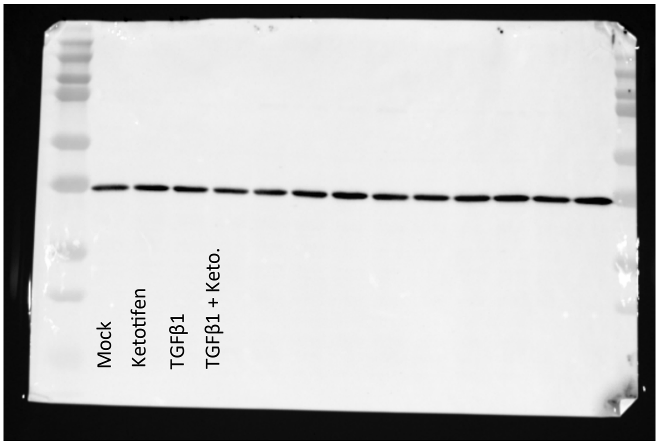

Supplementary Figure 8: Full western blot images with treatment groups annotated

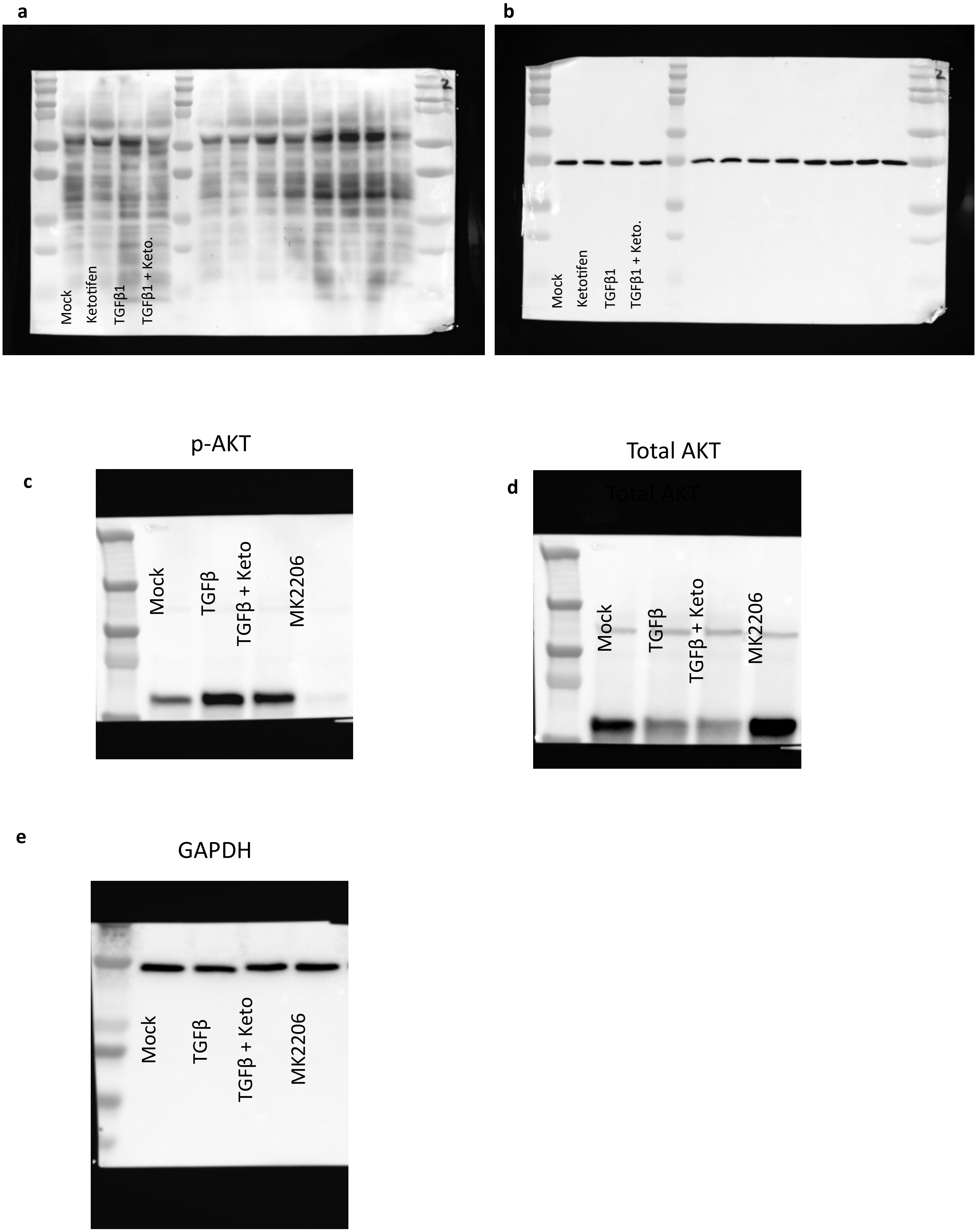

Supplement: Supplementary file 1 — Supplementary Information. [file 41598_2024_57776_MOESM1_ESM.pdf]
